# A dynamic model of COVID-19 infection quantifies the impact of preventive interventions on the infection of severely immunocompromised subjects in the United Kingdom

**DOI:** 10.1371/journal.pone.0341331

**Published:** 2026-02-23

**Authors:** Carmen Pin, Sylvia Taylor, Catia Ferreira, Sofie Arnetorp, Holly Kimko

**Affiliations:** 1 Systems Medicine, Clinical Pharmacology and Quantitative Pharmacology, Clinical Pharmacology and Safety Sciences, R&D, AstraZeneca, Cambridge, United Kingdom; 2 Medical and Scientific Affairs, Vaccines & Immune Therapies, Biopharmaceuticals, AstraZeneca, Cambridge, United Kingdom; 3 Medical and Scientific Affairs, Vaccines & Immune Therapies, Biopharmaceuticals, Wilmington, United States of America; 4 Health Economics, BioPharmaceuticals, AstraZeneca, Gothenburg, Sweden; 5 Systems Medicine, Clinical Pharmacology and Quantitative Pharmacology, Clinical Pharmacology and Safety Sciences, R&D, AstraZeneca, Gaithersburg, Maryland, United States of America; Iowa State University, UNITED STATES OF AMERICA

## Abstract

The disproportional risk of microbial infections affecting immunocompromised individuals underlines the critical need to develop effective infection preventive strategies. Using the COVID-19 pandemic as an example, we developed a mathematical model to evaluate interventions to protect severely immunocompromised (SIC) subjects against COVID-19. Predictions were well-aligned with UK available data for 2021 and 2022, and the model was used to retrospectively quantify the impact of preventive interventions in alternative scenarios during that period. Model simulations indicated that while the UK vaccination program reduced hospitalizations and deaths in the general population, SIC subjects remained at high risk of severe COVID-19. Simulated protective strategies, such as passive immunization, during seasonal SARS-CoV-2 peaks, showed potential to significantly reduce infection rates in this vulnerable group. We demonstrated the application of mathematical models to describe complex interactions among multiple dynamic processes and assess interventions to prevent disease transmission in both immunocompetent and immunosuppressed individuals.

## Introduction

At the close of 2019, China informed the World Health Organization (WHO) of a pneumonia of unknown cause, detected in the city of Wuhan in Hubei province [1]. On January 13^th^, 2020, the first case of novel coronavirus outside of China was confirmed [1]. On January 30^th^, 2020, the WHO declared that the infection outbreak constituted a Public Health Emergency of International Concern. On February 11^th^, the new disease was named COVID-19 and the novel coronavirus “severe acute respiratory syndrome coronavirus 2 (SARS-CoV-2)”. While 2021 was characterized by large peaks of infection all around the globe, during 2022, there was a considerable reduction in coronavirus disease hospitalisation and mortality in developed countries, which was attributable to broad access to COVID-19 vaccines, SARS-CoV-2 infection and reinfection acquired immunity and the emergence of attenuated SARS-CoV-2 variants [2].

The first dose of the vaccine against COVID-19 was deployed in the UK on December 8^th^ 2020 [3]. Vaccination of individuals in the UK was prioritized according to the risk of severe COVID-19, which was strongly determined by age and co-morbidities [4]. In January 2022, about a year time from the first dose, around 90% of people aged 12 years or older had been vaccinated at least once in the UK [5].

Despite the major reduction in COVID-19 hospitalisations and deaths, compared with the pandemic period, the high transmissibility of emergent variants and subvariants has led to high levels of circulating virus and the absolute number of deaths remains high [2]. Acquired immunity by COVID-19 vaccines and SARS-CoV-2 infection may be sufficient to safeguard most immunocompetent individuals against severe COVID-19. However, previous retrospective cohort studies show that fully vaccinated immunocompromised individuals remain at higher risk of hospitalisation, ICU admission, and death than the general population because they often have impaired immune responses to COVID-19 vaccination [6–9]. To address the vulnerability of immunocompromised individuals, preventive interventions different from vaccines have been developed to protect against SARS-CoV-2 infection and/or COVID-19 severe, and include the practice of preventive methods such as “cocoon vaccination” of routine contacts, anti-viral therapies, and passive immunization [10].

To evaluate how prophylactic strategies could protect immunocompromised individuals, we retrospectively quantify the impact of preventive measures on the COVID-19 pandemic by modelling SARS-CoV-2 infection dynamics across the UK in 2021 and 2022. The value of mathematical models to guide pandemic strategies has been previously highlighted [11]. Here, we developed a multicompartment Susceptible-Exposed-Infected-Resistant (SEIR) type model [12–18] that describes COVID-19 infection dynamics across severely immunocompromised (SIC) individuals and immunocompetent subjects in the UK population during 2021 and 2022. We modelled viral transmission and disease progression across multiple infection stages with age stratification and distinct immune states conferring protection against mild or severe disease. The framework incorporates variant-specific effects, seasonality, and both asymptomatic and symptomatic infection, and accounts for social events affecting mixing patterns as well as pharmaceutical and non-pharmaceutical interventions implemented in the UK during this period. The SIC population follows the “stringently defined immunocompromised” criteria from a prior report [9], encompassing individuals who, in the recent past, had one or more of the following conditions: moderate to severe primary immunodeficiency; active treatment with high-dose of corticosteroids or with non-corticosteroid immunosuppressive or immunomodulatory therapies; solid organ or stem cell transplant; solid or haematological malignancy on active treatment; specified haematological malignancies such as leukemias, lymphomas or multiple myeloma; and advanced or untreated HIV. We adopted a hybrid approach that couples ordinary differential equations with event-based scheduling to integrate discrete changes into otherwise continuous-time dynamics and align event timings with UK official data and published literature. We applied the model to analyse the pandemic trajectory and, in particular, to evaluate the outcomes of the SIC population under counterfactual scenarios.

## Results

### A mathematical model of COVID-19 transmission describes the pandemic dynamics across the UK population during 2021 and 2022 considering immunocompetent and SIC subjects

**Fig 1:**
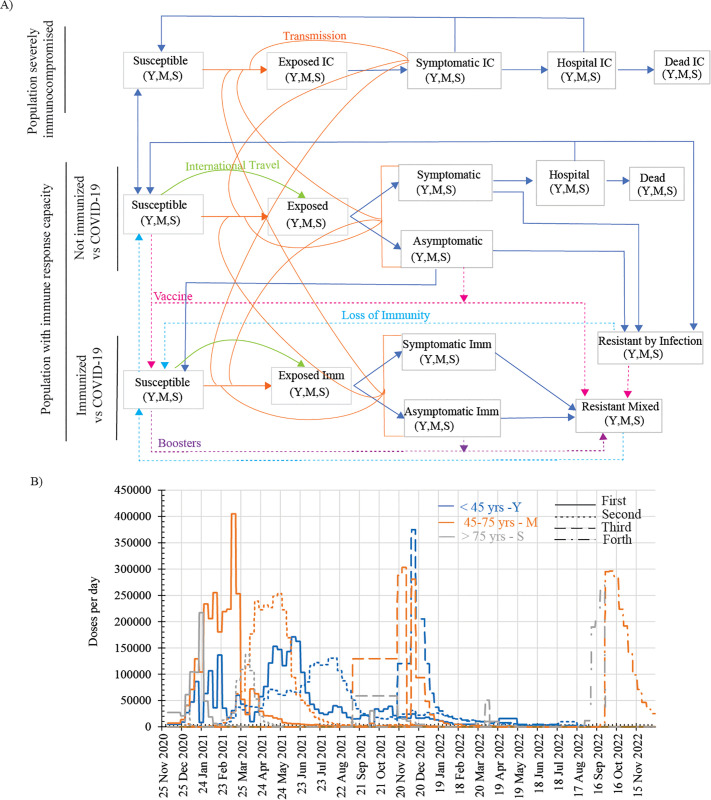
Modelling COVID-19 infection dynamics in the UK population from August 2020 to December 2022. **A)** Depiction of a model of the COVID-19 pandemic across a population partitioned into severely immunocompromised (SIC) individuals and immunocompetent subjects, who could be immunized subjects, by either infection or vaccination, and non-immunized against COVID-19 infection. The SIC population is further partitioned into susceptible (*Sus*^*(IC*^), incubating after exposure (*E*^*(IC*^), symptomatic (*Sym*^*(IC*^), hospitalized (*H*^*(IC*^), and dead (*D*^*(IC*^) subjects. The immunocompetent individuals naïve against COVID-19 infection are subdivided into susceptible (*Sus*), exposed **(*E*)**, infected symptomatic (*Sym*) and asymptomatic (*Asym*), hospitalized (*H*) and dead (*D*) compartments. After recovering from the infection, these subjects became immunized and resistant to both mild and severe disease (*RInf*). The COVID-19 immunized population does not comprise compartments for hospitalized or dead subjects, but includes susceptible to mild disease (*Sus*^*(Imm*^), incubating (*E*^*(Imm*^), symptomatic (*Sym*^*(Imm*^), and asymptomatic (*Asym*^*(Imm*^) subjects as well as resistant individuals to any form of the disease (*RMix*). Each compartment is subdivided in 3 age groups: < 45 (Y: young age), 45−75 (M: middle age) and >75 years (S: senior). COVID-19 transmission requires the contact between susceptible and infected subjects or could result from international travelling. Immunity can be acquired by vaccination or infection and may protect against mild and severe disease or against the severe form only. Loss of immunity is progressive with loss of protection against the mild form of the disease first. **B)** Rate of deployment of the first four sequential doses of COVID-19 vaccine to each age group in the UK according to published data by NHS England [32`]. The first dose is administered to susceptible (*Sus*) and asymptomatic (*Asym*) naïve subjects as well as to resistant individuals following infection (*RInf)*. Repeated doses or boosters are delivered to susceptible (*Sus*^*(Imm*^), asymptomatic (*Asym*^*(Imm*^) and resistant individuals (*RMix*) within the immunized population.

We have developed a model of the COVID-19 infection dynamics across the UK population during 2021 and 2022 that considers SIC individuals and immunocompetent subjects. The latter individuals are further subdivided in immunized subjects, by either infection or vaccination, and non-immunized against COVID-19. Numerous studies indicate that immunocompromised individuals exhibit significantly reduced immunogenicity to COVID-19 vaccines and infections, and this weakened immune response aggravates with increased severity of the immunocompromised condition [19–21]. Here, for the sake of simplicity, we assumed that SIC individuals are always symptomatic if infected and remain unprotected against any form of the disease, whether mild or severe, after infection or vaccination. On the other hand, immunocompetent subjects might be immunologically naïve against COVID-19 or immunized, by either infection or vaccination, and while protected against the severe form of the disease, they could be susceptible to COVID-19 infection with mild or no symptoms (Fig 1A). To capture age differences in the pandemic outcomes, we implemented three age groups which comprise subjects aged 45 or younger, between 45 and 75 and 75 or older (Fig 1A). Age ranges were selected to maximize within-group similarity in hospitalization and mortality outcomes, using reported data in the literature [22–25], while maximizing between-group contrasts (Supplementary Figure S1A in S1 File). Hereinafter, we referred to COVID-19 requiring hospitalization as severe disease and used the term mild disease for all other symptomatic conditions.

We consider that transmission of COVID-19 takes place after susceptible subjects encounter infected, either symptomatic or asymptomatic, immunosuppressed or immunocompetent subjects. Thus, transmission depends on the number of susceptible and infected subjects and the intensity of encounters between them. The infection transmission rate is further modulated by age differences in social activity [26] and overall health condition. Furthermore, COVID-19 variant-associated changes in the infection rate are implemented according to the reported timeline of the emergence of the alpha (B.1.1.7), delta (B.1.617.2) and omicron (B.1.1.529) variants (supplementary Figure S1B in S1 File) [27,28]. We also considered further modulation of the infection rate by COVID-19 seasonality [29,30] (supplementary Figure S1C in S1 File), international air travelling restrictions, periods of mandatory tests to enter the UK [31], isolation during symptomatic disease, government-imposed lockdown periods and school holiday in the UK. Age differences in hospitalization and death rates were also considered for both immunocompetent and SIC subjects.

We implemented the administration of the COVID-19 vaccine in our model using the actual vaccination deployment rates published by NHS England during 2021 and 2022 for each age group, which included up to four doses for subjects aged 45 and above [32] (Fig 1B). The first dose of vaccine conferred high level of protection against the severe form of the disease, but lower against the mild disease, as reported for vaccines used in the UK [33–35]. Repeated vaccine administration to already immunized subjects increased the protection against mild disease to 100% [33–35]. We assumed that efficient COVID-19 immunity was established 21 days after the first dose and 14 days after boosters’ administration as reported for the vaccine used in the UK [33–36]. We considered that immunity acquired by either infection or vaccination waned over time and resistant subjects to any form of the disease became susceptible first to the mild form [37–40] and later, if not receiving a booster or being infected, to the severe disease [41].

The model was calibrated using quantitative COVID-19 case data from the UK government and public sources (Table 1, Supplementary Table S1 in S1 File) from August 1^st^, 2020, to December 31^st^, 2022. Out of this specific context of application, i.e., country and period, model predictions are unreliable and similarly, jointly identified parameter values to describe high level data may be highly inaccurate when used in isolation. Supplementary Fig S2 in S1 File shows the predicted COVID-19 infection dynamics in each age group across all model compartments (Fig 1) in SIC and immunocompetent subjects during the study period.

**Table 1:** Description and identified values for model parameters and terms (parameter values are inter-dependent and meaningless out of the context of this model).

Term/Parameter	Value	Unit	Description
*n*	66.82x10^6^	subjects	Population in the UK [22]. Fixed during the simulation period.
[*f*_*Y*_*, f*_*M*_*, f*_*S*_]	[0.557, 0.358, 0.0085]	subjects	Fraction of UK population in age group Y (young age, < 45 years), M (middle age, 45–75 years) and S (senior, > 75 years) [22]. Fixed during the simulation period
$\alpha =\alpha_{A}+\frac{t^{hD}}{t^{hD}+t_{ChD}^{hD}}(\alpha_{D}-\alpha_{A})+\frac{t^{hO}}{t^{hO}+t_{ChO}^{hO}}(\alpha_{O}-\alpha_{D})$ where *t* = time		day^-1^	Function describing changes in the rate of successful infection (transmission) in susceptible individuals after contact with infected subjects according to the reported COVID-19 variants emergence timeline [27,28] (supplementary Fig 1A). In March-April 2021, when the delta variant became dominant, the value of *α* increased from *α*_*A*_ to *α*_*D*_, and this increased further to *α*_O_ in January 2022, when the omicron variant emerged
	*α*_*A*_ = 2.421 x10^-8^ *α*_*D*_ = 4.547 x10^-8^ *α*_*O*_ = 8.067 x10^-8^	day^-1^	Rate of successful infection in susceptible individuals after contact with infected subjects for alpha (B.1.1.7) (*α*_*A*_), delta (B.1.617.2) (*α*_*D*_) and omicron (B.1.1.529) (*α*_*O*_) variants. Calibrated with published UK government data [42] – specifically the number of hospitalized cases and deaths – recorded from August 1^st^, 2020, to December 31st, 2022 (supplementary Fig 1A).
	$t_{ChD}=279$ ** $t_{ChO}=506\;$ **	day	Midpoint in the period of emergence of the delta (B.1.617.2) (*t*_*ChD*_), May 7^th^, 2021; and omicron (*t*_*ChO*_) variant (B.1.1.529) dominance, December 20^th^, 2021 [9]
	$hD=\frac{log(\frac{\text{1}}{f_{Ref}}-1)}{log\left(\frac{t_{ChD}}{t_{ChD}+t_{Ref}/2}\right)}$ $hO=\frac{log(\frac{\text{1}}{f_{Ref}}-1)}{log\left(\frac{t_{ChO}}{t_{ChO}+t_{Ref}/2}\right)}$		Factors that recapitulate the dynamics of the variants emergence by regulating the change from *α*_*A*_ to *α*_*D*_ (*hD*) or from *α*_*D*_ to *α*_*O*_ (*hO*). The new variants achieved 95% dominance (*f*_*Ref*_ = 0.95) in a 45-day period (*t*_*Ref*_ = 45 days) [27,28].
env(*T*)	$env\left(T\right)=\frac{2\cdot T_{50}}{T_{50}+T}$ where, *T*_*50*_ = 20 *T*= temperature (°C)		Function implementing COVID-19 infection seasonality [29,30] that regulates the dependence of the rate of infection on environmental temperature (monthly averages) as reported for Heathrow climate station by the UK Met Office from August 1^st^, 2020 to December 31st, 2022 [48] (Supplementary Fig 2B). Calibrated with published UK government data [42] – specifically the number of hospitalized cases and deaths – recorded from August 1^st^, 2020 to December 31st, 2022 (Fig 2A).
*λ*	0.5 if 96 ≥ t** ≤ 123 (Lockdown 20) 0.7 if 157 ≥ t** ≤ 289 (Lockdown 21) 0.5 if 616 ≥t** ≤ 631 (Easter 22) 0.8 if 713 ≥ t** ≤ 758 (Summer 22) 1 otherwise (no effect)		Factor that accounts for changes in population social activity or encounters leading to viral transmission, during lockdown periods and school holiday in the UK. During this study timeline, the UK government imposed two national lockdown periods between November 5^th^ and December 2^nd^ of 2020, and between January 5^th^ and May 17^th^ of 2021. In 2022, with social restrictions lifted, we considered a reduction in transmissions due to school holiday during the Easter break from April 9^th^ to April 23^rd^ and the summer holiday from July 15^th^ to September 15^th^. Calibrated with published UK government data [42] – specifically the number of hospitalized cases and deaths – recorded from August 1^st^, 2020 to December 31st, 2022. (Fig 2A).
[*σ*_*Y*_*, σ*_*M*_*, σ*_*S*_]	[0.65, 0.11, 0.24]		Factor that modulates infection transmission for each age group (Y, M, S), likely reflecting age differences in social activity [26] and overall health of subjects. Higher values indicate higher transmission. Calibrated to reflect the differences in the proportion of infection cases reported within each age group from August 1^st^, 2020, to December 31^st^, 2020, by the UK office for National Statistics [47] before the age prioritization vaccination campaign started in the UK (Fig 2A).
*θ*	0.01		Quarantine factor that decreases encounters with other subjects of symptomatic individuals observing isolation. This value is assumed.
*τ*	1/5	day^-1^	Rate of viral incubation prior to infection [15]
*f*_*Asym*_	0.4		Fraction of infected subjects without symptoms [49]
*ρ*_*Sym*_	1/10	day^-1^	Rate of infection clearance of non-hospitalized symptomatic subjects. Estimated according to the expected time of infectious virus shedding by infected individuals [49,50].
*ρ*_*Asym*_	1/10	day^-1^	Rate of infection clearance of asymptomatic subjects. We assumed the same rate of infection clearance for asymptomatic subjects as for non-hospitalized symptomatic subjects. Viral dynamics in asymptomatic patients is poorly documented [51] with no significant differences reported between the viral load of symptomatic and asymptomatic patients [49,52].
[*ω*_*Y*_*, ω*_*M*_*, ω*_*S*_]	*ω* = 0.025 *ω* x [0.01, 0.25,0.74]	day^-1^	Rate of hospitalization of symptomatic subjects for each age group. Calibrated with published UK government data [42] – specifically the number of hospitalized cases and deaths – recorded from August 1^st^, 2020 to December 31st, 2022 (Fig 2A). The age-dependent rates were calculated considering the age variation in hospitalization cases reported by the centre for disease control and prevention [25] and by a study across hospitals in the UK [24] from August 1^st^, 2020, to December 31st, 2020 before the age prioritization vaccination campaign started in the UK. (Fig 2B)
*ν*	2/3 x 1/7	day^-1^	Rate of recovery of hospitalized subjects. The estimation of the rate of recovery in hospitalized patients is based on the analysis of data extracted from hospitals in the UK, which suggests that about two thirds of the patients recover after spending about 7 days (median value) in hospital [24].
$\delta =\delta_{A}+\frac{t^{hD}}{t^{hD}+t_{\delta ChD}^{hD}}(\delta_{D}-\delta_{A})+\frac{t^{hO}}{t^{hO}+t_{\delta ChO}^{hO}}(\delta_{O}-\delta_{D})$ where *t* = time		day^-1^	Function describing changes in the rate of death in hospitalized individuals infected with either alpha (B.1.1.7), delta (B.1.617.2) or omicron (B.1.1.529) variants, according to COVID-19 variants emergence timeline [27,28]. In March-April 2021, when the delta variant became dominant, the value of *δ* decreased from *δ*_*A*_ to *δ*_*D*_, and this decreased further to *δ*_O_ in January 2022, when the omicron variant emerged.
	*δ*_*A*_ = 0.0680 *δ*_*D*_ = 0.0450 *δ*_*O*_ = 0.0250	day^-1^	Rate of death in hospitalized individuals infected with either alpha (B.1.1.7) (*δ*_*A*_), delta (B.1.617.2) (*δ*_*D*_) or omicron (B.1.1.529) (*δ*_*O*_) variants. Calibrated with published UK government data [42] – specifically the number of hospitalized cases and deaths – recorded from August 1^st^, 2020 to December 31st, 2022 (Fig 2A).
	$t_{\delta ChD}=279+20$ ** $t_{\delta ChO}=506+20\;$ **	day	Consolidation date of the delta (B.1.617.2) (*t*_*ChD*_) and omicron (*t*_*ChO*_) variant (B.1.1.529) [9] as cause of death. We implemented a 20-day delay between the adjustment of infection and death kinetics for the new variant.
	$hD=\frac{log(\frac{\text{1}}{f_{Ref}}-1)}{log\left(\frac{t_{\delta ChD}}{t_{\delta ChD}+t_{Ref}/2}\right)}$ $hO=\frac{log(\frac{\text{1}}{f_{Ref}}-1)}{log\left(\frac{t_{\delta ChO}}{t_{\delta ChO}+t_{Ref}/2}\right)}$		Factor that recapitulates the dynamics of the variant emergence by regulating the change from *δ*_*A*_ to *δ*_*D*_ (*hD*) or from *δ*_*D*_ to *δ*_*O*_ (*hO*). A new variant achieves 95% dominance (*f*_*Ref*_ = 0.95) in a 45-day period (*t*_*Ref*_ = 45 days). [9]
[*δ*_*Y*_*, δ*_*M*_*, δ*_*S*_]	*δ* x [0.1, 0.35, 0.55]	day^-1^	Rate of death of hospitalized subjects for each age group. Calibrated to reflect the differences in the proportion of infection cases reported within each age group from August 1^st^, 2020 to December 31^st^, 2020 by several sources [23,53] before the age prioritization vaccination campaign started in the UK (Fig 2B).
[effi_1sev_, effi_1mild_, effi_Boo_]	[1, 0.70, 1]		Factor describing vaccine efficacy. The first dose, or dose in Covid-19 naïve individuals, was assumed to provide protection to 100% subjects against severe disease and 70% protection against mild disease. Subsequent doses in already immunized individuals, hereby referred to as boosters, increased the protection against mild disease to 100% [33–35].
vac_1_ (*X*_*i*_)	$=\frac{X_{i}}{Sus_{i}+Asym_{i}+RInf_{i}}DR_{i}$ *X*_*i*_*={Sus*_*i*_*, Asym*_*i*_*, RInf*_*i*_*}* *DR*_*i*_ = Interpolation(t** − 21, data FirstDose/day) *i* ={*Y*, *M*, *S*}	subjects/day	Rate of deployment of the first vaccine dose to the COVID-19 naïve population. The deployment rate for each age group, *DR*_*i*_ with i={*Y, M, S*}, is interpolated from the actual vaccination deployment rates published by NHS England (Fig 1B) [32`]. We assumed that efficient COVID-19 immunity is established 21 days after administration [33–36]. Doses are distributed proportionally to the number of susceptible (*Sus*_*i*_), asymptomatic (*Asym*_*i*_) and resistant by infection (*RInf*_*i*_) subjects in each age group.
boost (*X*_*i*_)	$=\frac{X_{i}}{Sus_{i}^{(Imm}+Asym_{i}^{(Imm}+RMix_{i}}DR_{i}$ *X*_*i*_*={Sus*^*(Imm*^_*i*_*, Asym*^*(Imm*^_*i*_*}* *DR*_*i*_ = Interpolation(t** − 14, data Boosters/day) *i* ={*Y*, *M*, *S*}	subjects/day	Rate of deployment of doses in already immunized subjects by vaccination. The deployment rate for each age group, *DR*_*i*_ with i={*Y, M, S*}, is interpolated from the actual vaccination deployment rates published by NHS England (Fig 1B) [32]. We assumed that efficient COVID-19 immunity is established 14 days after administration [33–36]. Doses are distributed proportionally to the number of immunized susceptible (*Sus*^*(Imm*^_*i*_), asymptomatic (*Asym*^*(Imm*^_*i*_) and resistant subjects (*RMix*_*i*_) in each age group.
*ϕ*	1/(4.5x30) 5/(4.5x30) If (506 + 60 < *t* < 506 + 65)	day^-1^	Rate of loss of immunity against mild disease [37–40]. We considered that immunity developed against the delta variant provided limited protection against the omicron variant infection as previously reported [45] and implemented an acute but brief increase in the transfer of subjects from resistant (resistant mixed compartment) to “susceptible to mild disease” (susceptible immunized population) state, 60 days after the omicron variant emergence, when it became dominant – [9]. Calibrated with published UK government data [42] – specifically the number of hospitalized cases and deaths – recorded from August 1^st^, 2020 to December 31st, 2022 (Fig 2A).
*ϕ*_*SusImm*_	1/(3x4.5x30)	day^-1^	Rate of loss of immunity loss against severe disease. We assumed that immunity against severe disease lasts 3-fold longer than against mild disease. Published reports indicate that reinfection may occur after few moths but with 1/3 hospitalizations [41]
*κ*	0 if t** < 366 0.0004 x 0.5 x 250x10^6^/(365x66.82 x10^6^) if 366 ≤ t** < 594 0.002 x 0.5 x 250x10^6^/(365x66.82 x10^6^) if t** ≥594	day^-1^	Rate of infection associated with international travelling by air, assuming that international passenger movements in UK airports returned to pre-COVID levels (250 million/year [28]) on August 2^nd^, 2021. Previous reports suggest a risk of transmission associated with air travel of 0.04%, when passengers take a rapid antigen COVID-19 detection test within 72-hour prior to the flight, and of 0.2% when passengers are not tested [31]. COVID-19 testing stopped being mandatory to enter the UK on March 18^th^, 2022.
*nIC*	0.007 x *n*	subjects	Number of SIC subjects in the UK as per published reports [9]. Fixed during the simulation period.
$\left[f_{Y}^{(IC},f_{M}^{(IC},f_{S}^{(IC}\right]$	[0.271, 0.522, 0.207]		Fraction of SIC subjects in age group Y (young age, < 45 years), M (middle age, 45–75 years) and S (senior, > 75 years) (estimated from the age distribution of selected long-term health conditions published by the UK Office for National Statistics [54]). Fixed during the simulation period.
[*β*_*Y*_*, β*_*M*_*,, β*_*S*_]	$\left[f_{Y}^{(IC},f_{M}^{(IC},f_{S}^{(IC}\right]$*nIC/180	subjects/day	Renewal rate of the SIC population. Denotes the in- and out-flow of SIC subjects in each age group required to maintain the size of this population. Assumed.
*ξ*	0.00605		Factor that modulates infection transmission in immunocompromised individuals, likely reflecting decreased social activity, overall health condition of these subjects or protective strategies applied solely to SIC subjects. Impact of age differences in social activity or overall health condition on infection rate was disregarded in this group [43]. Calibrated with published data [9] on hospitalization percentage (Fig 2D).
$\left[\omega_{Y}^{(IC},\omega_{M}^{(IC},\omega_{S}^{(IC}\right]$	*ω* x 16 x [0.10, 0.35, 0.55]	day^-1^	Rate of hospitalization of infected SIC for each age group. Reported to be sixteen-fold higher than the rate of immunocompetent individuals [20]. Age distribution calibrated with published data [20] (Fig 2E).
$\left[\delta_{Y}^{(IC},\delta_{M}^{(IC},\delta_{S}^{(IC}\right]$	1.02 x [*δ*_*Y*_*, δ*_*M*_*, δ*_*S*_]	day^-1^	Rate of death of hospitalized SIC individuals for each age group. It changes with the COVID-19 variant and increases in older people recapitulating changes in the death rate of the immunocompetent population. Calibrated with published data [9] on percent of SIC dead subjects within total deaths (Fig 2D).
*ε*	[0, 0.25, 0.5, 0.75, 1] if 440 ≥ t** ≤ 622 or t** ≥ 805 0 Otherwise		Protection (0, 25, 50, 75 or 100%) conferred to the SIC population by preventive interventions from October 15^th^, 2021, to April 15^th^, 2022, and from October 15^th^, 2022, to end of the study in December 2022 during COVID 19 infection peaks.

*s.e is standard error

**Day 0 is August 1^st^, 2020

### Model predictions are aligned with a variety of observations gathered in the UK during 2021 and 2022, including SIC subjects

**Fig 2:**
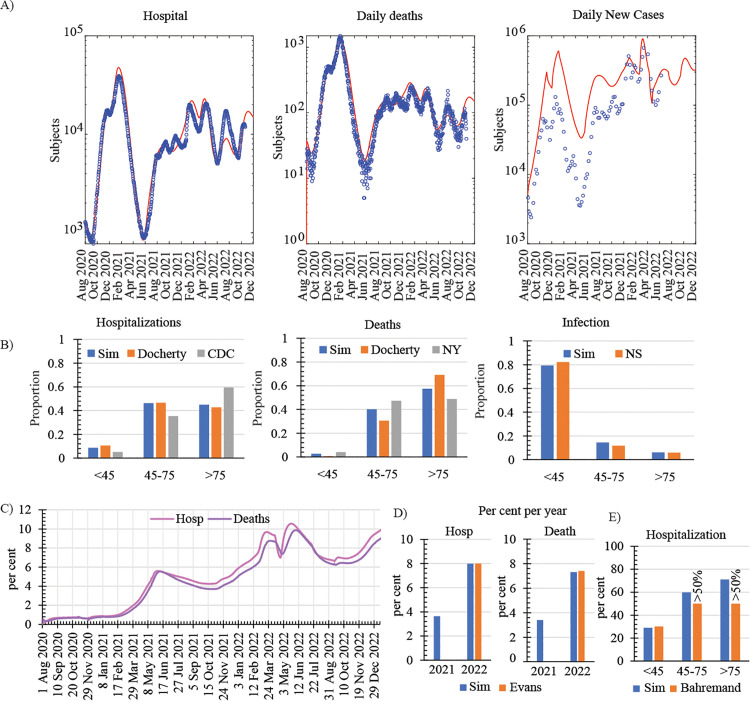
Comparison between model predictions and available COVID-19 pandemic data. **A)** Comparison of reported hospitalized cases, daily deaths, and daily new cases of infection (symbols) reported by the UK government [42] with model predictions (lines) from Aug 2020 to December 2022; **B)** Simulated and observed age distribution of infected and hospitalized cases and deaths. Simulations were performed from Aug 1^st^, 2020, to Nov 30^th^, 2021, before any vaccine deployment in the UK. Simulated age distributions (Sim) were compared with data reported by the UK office for National Statistics (NS) [47], Docherty *et al.* for UK hospitals [24], the centre for disease control and prevention (CDC) [25] and the city of New York [23]; C-D) Simulated percentage of hospitalized cases and deaths in the SIC population relative to the total hospitalized cases and deaths in the UK from Aug 2020 to December 2022 per day (C) and per year **(D)**. The simulated percentage per year, 2021 and 2022, is plotted together with data reported by Evans *et al*. [9]; **E)** Simulated and reported by Bahremand *et al*.[20] hospitalization rates relative to COVID-19 cases for each age group in the SIC population between January 7, 2022 and March 14, 2022.

Fig 2A shows that the simulated results for overall hospitalized subjects and daily deaths are in good agreement with data reported by the UK government from August 1^st^, 2020, to December 31^st^, 2022 [42]. At the beginning of this period and during most of 2021, the prediction for new cases of infection is higher than the number of COVID-19 positive cases recorded by the UK government [42] (Fig 2A). This could reflect the progressive building of the testing capacity and strategy to meet the UK demand. Similar disagreements on estimated and reported infection cases during this period have been previously described [13,14].

For the immunocompetent population, we assumed that the higher infection rates reported in young subjects, and recapitulated by the model (Fig 2B), could be associated with more intense social activity in this age group [26]. We also observed that the model factor modulating the effect of the age on the infection rate was higher in senior subjects (*σ*_*S*_= 0.24, Table 1) than in the middle age group (*σ*_*M*_= 0.11, Table 1), which could be explained by poorer overall health in subjects aged 75 and above leading to greater susceptibility to infection. Thus, for the immunocompetent population, our simulations and available data suggest that the youngest group was the main age group harbouring the virus and, mainly when asymptomatically infected, responsible for viral transmission (Fig 2B). On the other hand, in agreement with available data (Fig 2B), the model predicts that hospitalisations and deaths mainly occur in subjects over 45 years, (Fig 2B). In particular, subjects aged 75 and above exhibit the highest proportion of hospitalization cases and deaths, although they exhibit lower infection rates, and this observation is well aligned with model predictions (Fig 2B).

To parametrize our simulated SIC population, we use the reported quantitative data on “stringently defined immunocompromised” subjects by Evans *et al*. [9]. In alignment with the data in that report, we assumed that this group represents 0.7% of the UK population. We considered that the impact of age on the risk of infection among SIC individuals could be neglected as previously reported [43]. For the SIC population, we introduced an additional parameter to account for the reduced infection rate resulting from their decreased social activity or additional protective measurements. This adjustment reflects the recommended shielding measures, which encourage these individuals to minimize contact with others. Moreover, we considered a higher overall risk of hospitalization in infected SIC individuals relative to immunocompetent subjects and higher likelihood of hospitalisation in older individuals as previously reported [20]. Having implemented these details in our model, our simulated hospitalization and death percentages of SIC subjects, relative to the hospitalization and death cases in the total population, respectively, were in good agreement with published data [9] (Figs 2C-2D). Notice that the smaller hospitalization and death percentages of SIC subjects in 2021, compared to 2022, are due to considerably larger numbers of clinical cases in the immunocompetent population in 2021 when the population immunity against SARS-CoV-2 was relatively low. Similarly, the age associated hospitalization rates were in good alignment with published data of SIC subjects [20] (Fig 2E).

Altogether, we have developed a model of the dynamics of COVID-19 infection across the UK population during 2021 and 2022, able to recapitulate available data for the overall population as well as for SIC subjects.

### Model sensitivity analysis highlights the impact of asymptomatic infections on viral transmission and the influence of SIC-specific protective strategies on outcomes in SIC subjects

We evaluated the sensitivity of the model state variables to key parameters governing infection dynamics and assessed effects separately in the immunocompetent and SIC populations (Supplementary Table S2 in S1 File). The most influential parameter in the immunocompetent population, and highly influential in the SIC population, was *f*_*Asym*_, the fraction of infections that are asymptomatic. Increases in the value of this parameter were associated with widespread rises in state variables, consistent with intensified viral transmission and circulation, and underscored the central role of asymptomatic infection in propagation. The incubation and transmission rates exerted likewise a strong effect on the model outcomes; higher values similarly amplified viral circulation. The infection recovery rate (or infection clearance) for asymptomatic cases showed a stronger impact than the recovery rate for symptomatic cases, further supporting asymptomatic individuals as key drivers of transmission (Supplementary Table S2 in S1 File). Hospitalization and mortality rates had had their largest effects on the SIC population, which, by model designed, is assumed not to develop immunity and is therefore more sensitive to changes in parameters linked to severe disease.

The parameter with the greatest impact on the SIC population was that modulating SIC-specific infection transmission (Supplementary Figure S3, Supplementary Table S2 in S1 File). Infection transmission in SIC subjects differs from the immunocompetent population due to restrictions in social activity, diminish overall health condition or specific protective measurements applied to this population to modify transmission. This parameter exerted a strong influence on outcomes (Supplementary Figure S3, Supplementary Table S2 in S1 File) which indicated that SIC-specific protective measures could have substantial effects on this population. Notably, increasing values of this parameter heightened viral circulation exclusively within the SIC population.

### Hospitalization and death of immunocompetent subjects are predicted to have been strongly reduced by the vaccination program in the UK mainly during 2021

We next aim to use the model to quantify the impact of the vaccination strategy in the UK. To that end, we simulated scenarios considering no vaccination of i) any subject, ii) the young group iii) the middle age group or iv) the senior group during 2021 and 2022. We compared the simulated results with the predicted pandemic outcomes by the original model described above and validated with existing data.

**Fig 3:**
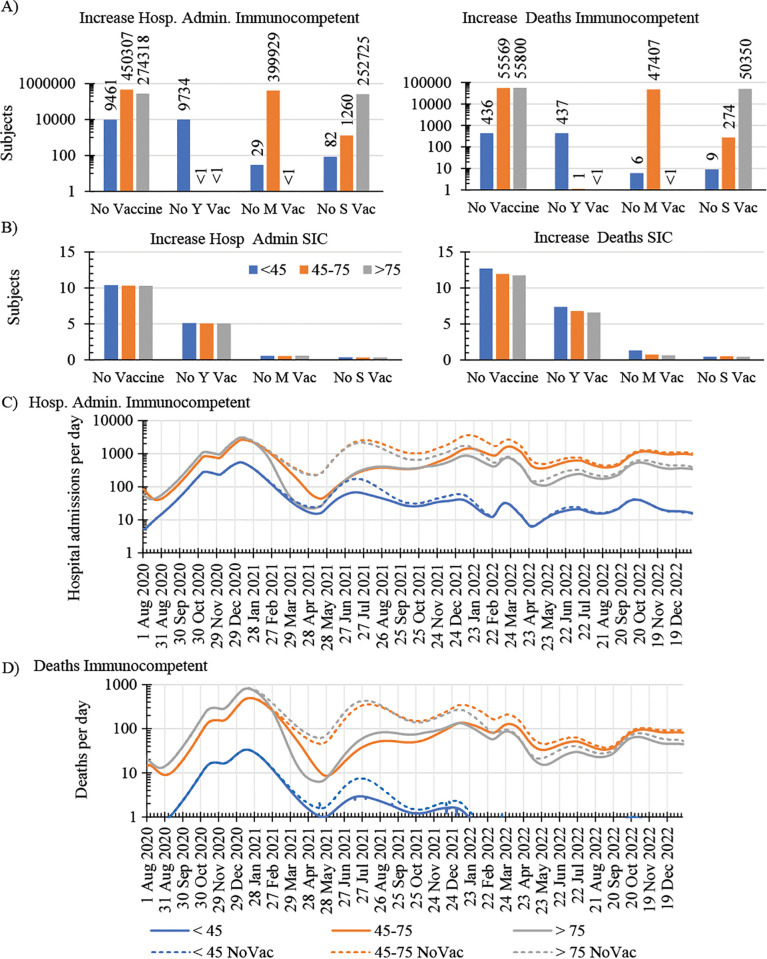
Simulated impact of vaccination on COVID-19 dynamics across the immunocompetent and SIC population in the UK. Predicted increase in the number of hospital admissions and deaths of immunocompetent (A) and SIC (B) subjects under simulation conditions considering no vaccination of i) any subject, ii) young, Y, iii) middle age, M, or iv) senior, S, subjects during 2021 and 2022. Daily hospital admissions (C) and deaths (D) of immunocompetent subjects under the vaccination programme implemented in the UK according to published data by NHS England [32`] (solid line), which is described in Fig 1B, and under simulated conditions assuming no vaccination of any subject during 2021 and 2022 (dashed line).

For the scenario considering no vaccination of any subject, our model predicted an increase of more than 110,000 deaths and 730,000 hospitalization admissions in the immunocompetent population during 2021 and 2022 (Fig 3A). The exclusion from the vaccination campaign of either the middle age or senior group would have resulted in an increase of about 400,000 or 250,000 hospital admissions, respectively, and about 50,000 deaths in either case. Missing the vaccination of the young group would have had a comparatively minor increase of 500 deaths and 10,000 hospital admissions (Fig 3A). On the other hand, the vaccination of immunocompetent subjects was predicted to have a minor effect on the pandemic outcomes of SIC subjects with a very minor increase of about 0.7% deaths and 0.06% hospital admission cases in the no vaccination scenario (Fig 3B). This result suggests that despite the vaccine-induced disruption of the infection cycle in the general population, the level of circulating virus remained sufficiently high to infect SIC subjects and changes in disease outcomes for SIC subjects associated with vaccination were predicted to be negligible.

Our model assumes that SIC subjects remain unprotected against COVID-19 infection after vaccination or after infection. While immunocompromised individuals are known to have a significantly reduced immune response compared to immunocompetent individuals, they may still exhibit some degree of protection following vaccination [19–21]. For simplicity, we assumed individuals with severely immunocompromised conditions are not able to mount efficient immune responses. This assumption facilitated the assessment of how increased immunity, driven by vaccination, of the general population, could impact on the pandemic outcomes of the SIC population, without confounding from their own immune responses. In addition, there is a lack of accurate quantitative information on the immune response of SIC subjects required for a precise estimation of the response to vaccination or infection on SIC subjects. Nevertheless, this assumption may affect the absolute outcomes of the SIC population under counterfactual simulation scenarios. Accordingly, those numbers should be interpreted as illustrative and used for comparative purposes only.

Figs 3C-3D shows that the differences in hospital admissions and deaths between the no vaccination scenario and the original model, describing the actual pandemic outcomes on immunocompetent subjects, are more apparent in 2021 than in 2022 for all age groups. This indicates that the vaccination of immunocompetent subjects had the greatest impact in 2021 before the so-called herd immunity was achieved in the population in 2022 (Figs 3C-3D).

All together our simulation results suggest that the vaccination strategy deployed in the UK led to the early increase of the population immunity and mainly during 2021 prevented a considerable increase of the already high reported number of deaths and hospital admissions in the UK during the COVID-19 pandemic.

### Preventive protective interventions during COVID-19 peak of infections could strongly reduce hospitalization and deaths of SIC subjects

We further used the model to investigate the impact of preventive interventions, such as passive immunization, conferring protection to SIC subjects against COVID-19 infection. For this purpose, we shortened the study period from April 1^st^, 2021, to December 31^st^, 2022, and simulated scenarios including preventive passive immunization during the infection peaks from October 15^th^, 2021, to April 15^th^, 2022, and from October 15th, 2022, to the end of the study period. Thus, we avoided the inclusion of the extremely large peak of infection recorded at the end of 2020-beginning 2021 associated with the extremely low initial population immunity and that could lead to an exaggeration of the impact of preventive interventions on clinical outcomes in SIC subjects.

We simulated 4 possible scenarios comprising interventions conferring 10%, 25% 35% and 50% protection against COVID-19 infection during the infection peak periods mentioned above. Protection levels in our simulations refer to the percentage of individuals protected against infection. We compared the simulated results in SIC subjects with the predicted outcomes by the original model calibrated with existing data and predicting the actual pandemic dynamics.

**Fig 4:**
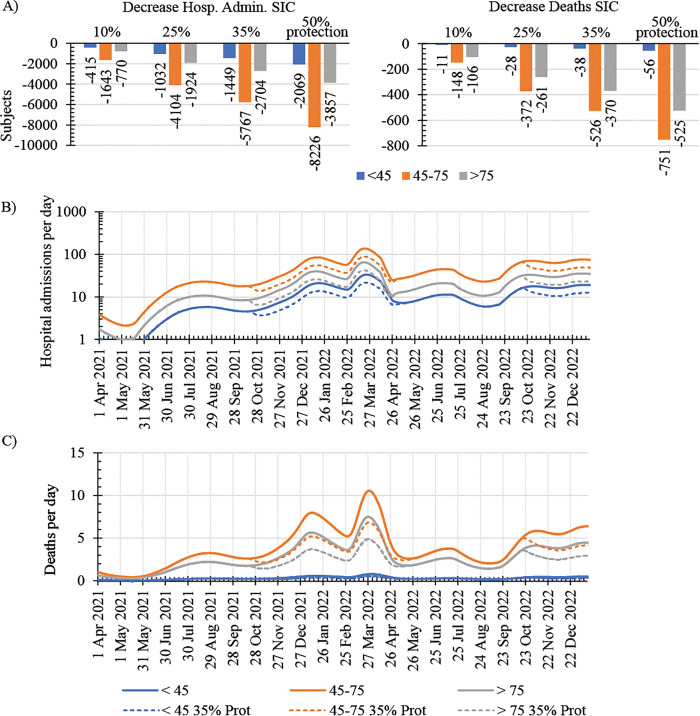
Simulated impact of preventive interventions during COVID 19 infection peaks in the SIC population. **A)** Predicted decrease in the number of hospital admissions and deaths of SIC subjects, accumulated from April 2021 to December 2022, under simulated conditions considering preventive interventions that confer 25, 50, 75 and 100% protection to the SIC population from October 15^th^, 2021, to April 15^th^, 2022, and from October 15^th^, 2022, to end of the study in December 2022, during the epidemiological infection peaks. Daily hospital admissions (B) and deaths (C) of SIC individuals with (dashed line) and without (solid line) considering preventive interventions conferring 75% protection against COVID-19 infection during the epidemiological infection peaks from October 15^th^, 2021, to April 15^th^, 2022, and from October 15^th^, 2022, to end of the study in December 2022.

Our original model calibrated with existing data predicted about 4,400 deaths and 44,000 hospital admissions of SIC subjects from April 2021 to Dec 2022. In our simulation scenario with preventive interventions conferring 35% protection against COVID-19 infection, these numbers decreased by approximately 900 and 10,000, respectively, and further by about 1300 and 14,000, respectively, when the simulated preventive interventions achieved 50% protection against infection (Fig 4A). Of note, the efficacy of our simulated preventive interventions was age-independent, and its relative impact was consistent across all age-groups. The greater absolute decrease in clinical cases predicted for the middle age group was the result of a greater number of casualties in this age group. The temporal dynamics of the simulated clinical outcomes suggests a rapid response to preventive interventions with considerable decreases in hospital admissions and deaths of SIC subjects during the intervention periods (Figs 4B-4C).

Our simulation results indicate that protective interventions, such as passive immunization, preventing COVID-19 transmission to SIC subjects during infection peaks could significantly mitigate the risk of severe COVID-19 in this population.

## Discussion

We developed a mathematical model of the COVID-19 pandemic with emphasis on its application to understand the impact of preventive strategies on SIC subjects. To that end, we focussed on recapitulating the observed disease dynamics in the UK population during 2021 and 2022 and performed simulations in alternative scenarios. To achieve a good representation of available data on the general population and SIC subjects in the UK, our model assumed that SARS-CoV-2 transmission rates were affected by age differences on social activity and overall health condition, emerging new variants, seasonality and population-wide social and travelling restrictions, and implemented the vaccination rates published by NHS England [32]. Furthermore, we considered that immunocompetent subjects, following vaccination or infection, could become protected against COVID-19 mild and severe disease or only to the severe disease, while SIC subjects did not develop any effective protection after vaccination or infection. This level of granularity was sufficient to achieve a satisfactory representation of the observed disease dynamics and enabled the simulation of alternative prophylactic strategies during 2021 and 2022 in the UK.

In agreement with previous mathematical modelling studies [44], our simulations indicate that the vaccination strategy deployed in the UK was an efficient intervention to control COVID-19 by enhancing the population immunity while preventing severe disease cases, leading to a faster containment of the pandemic. However, immunocompromised individuals remain highly disproportionately impacted by COVID-19, despite high rates of vaccination, accounting for more than 20% of COVID-19 hospitalisations, ICU admissions, and deaths in England [9]. Our results indicate that preventing measurements delivering 25%, 35% or 50% protection to the SIC population could reduce hospital admissions and deaths by 15%, 22% and 31%, respectively, when delivered during the seasonal peak of SARS-CoV-2 infection. These results emphasize the continuous need for the development and implementation of innovative strategies to safeguard immunocompromised populations.

Given the complexity of SARS-CoV-2 infection dynamics, mathematical models are highly suitable to investigate the impact of preventive strategies, therapeutics and alternative scenarios on the infection cycle and disease and, hence, assess outcomes for vulnerable populations, the pressure on the healthcare system, and the overall death toll. However, it is worth noticing that the forecasting performance of this type of models is limited by the uncertainty to predict future events such as the emergence of new variants or socioeconomic political events affecting the dynamics of the infection cycle. In this study, we developed a model to characterize the COVID-19 pandemic in the UK during 2021 and 2022. Model hypotheses could be adjusted, and regional or period-specific modifications could be implemented to help evaluate prophylactic and therapeutic strategies aimed at safeguarding global populations against COVID-19 and other viral or bacterial infections.

## Materials and methods

### Model description

Ou model describes the temporal dynamics of COVID-19 infection across a population partitioned into compartments. A high-level partition divides the population into SIC individuals, unable to develop any immune response against COVID-19, and immunocompetent subjects. The latter population is further subdivided in immunized subjects, by either infection or vaccination, and non-immunized against COVID-19 infection (Fig 1A). We neglected the impact of births and deaths on the dynamics of the immunocompetent population but considered the in- and out-flow of immunocompromised subjects required to balance the size of this population.

The SIC population is further partitioned into susceptible (*Sus*^*(IC*^), exposed in incubation stage (*E*^*(IC*^), symptomatic (*Sym*^*(IC*^), hospitalized (*H*^*(IC*^), and dead (*D*^*(IC*^) subjects (Fig 1A). Regarding the immunocompetent individuals naïve against COVID-19 virus, we assumed that they are susceptible to the mild and severe form of the disease and subdivided this population into susceptible (*Sus*), exposed in incubation stage (*E*), infected symptomatic (*Sym*) and asymptomatic (*Asym*), hospitalized (*H*) and dead (*D*) compartments. If recovering from the infection, these subjects became immunized against COVID-19 and resistant to both mild and severe disease (*RInfc*).

The COVID-19 immunized population does not include compartments for hospitalized or dead subjects, but includes susceptible to mild disease (*Sus*^*(Imm*^), incubating (*E*^*(Imm*^), symptomatic (*Sym*^*(Imm*^), and asymptomatic (*Asym*^*(Imm*^) subjects as well as resistant individuals to any form of the disease (*RMix*). (Fig 1A). Thus, there are two compartments of resistant subjects to mild and severe disease, one of them, *RInf,* comprises non-vaccinated individuals, while the other, *RMix*, includes mostly vaccinated individuals. This partition of the resistant individuals aimed to achieve a more realistic apportion of vaccine first doses to the population during 2021 and 2022 in the UK [32`]. Each compartment is further subdivided in three age groups that include subjects aged 45 or younger (young, *Y*), between 45 and 75 (middle, *M*) and 75 or older (senior, *S*) (Fig 1A).

In alignment with UK vaccination protocols published by NHS England during 2021 and 2022, [32] (Fig 1A), we simulated the administration of the first dose of the vaccine to non-immunized subjects, who could be susceptible or undergoing their first asymptomatic infection, and to subjects already immunized after recovering from infection (*RInf*). Boosters were delivered to the immunized population including susceptible, asymptomatic infected and resistant subjects after infection or vaccination (*RMix)*.

We implemented the infection dynamics of new COVID-19 variants by changing the rate of infection and death according to the reported timeline of the emergence of the alpha (B.1.1.7), delta (B.1.617.2) or omicron (B.1.1.529) variants (supplementary Figure S2B in S1 File) [27,28] from August 2020 to December 2022. Furthermore, we also considered that the immunity developed against the delta variant offer limited protection against the omicron variant infection as previously reported [45] and implemented a reduction in the number of resistant subjects to the mild disease after the emergence of the omicron variant.

The rate of infection was further modulated with various terms that quantified the impact of social activity [26] and overall health condition according to the subjects age, COVID-19 infection seasonality [29,30] (supplementary Figure S2C), international air travelling restrictions with periods of mandatory tests to enter the UK [31], isolation during symptomatic disease, lockdown periods and school holiday in the UK.

For SIC subjects, the age-independent factor that modulates infection transmission was calibrated using published data [9] on hospitalization cases of immunocompromised individuals (Fig 1F). We also considered that the rate of hospitalization of infected SIC individuals was sixteen-fold higher than that of immunocompetent individuals [20] and it was age-dependent, with higher likelihood of hospitalisation in older individuals as previously reported [20]. The death rate of hospitalized SIC subjects was calibrated using published data (Fig 2D) [9] resulting in slightly different values, about 10% lower, from those of immunocompetent subjects (Table 1). The remaining model parameters shared the values identified for the immunocompetent population (Table 1).

The model is described by the following system of ordinary differential equations (ODEs):

### SIC population


$$\begin{array}{cc} \frac{dSus_{i}^{(IC}}{dt}=\; & -\alpha env\left(T\right)\lambda \theta \frac{\left(\sigma_{Y}Sym_{Y}+\sigma_{M}Sym_{M}+\sigma_{S}Sym_{S}\right)}{n}\xi \varepsilon Sus_{i}^{(IC}\\ \; & -\alpha env\left(T\right)\lambda \frac{\left(\sigma_{Y}Asym_{Y}+\sigma_{M}Asym_{M}+\sigma_{S}Asym_{S}\right)}{n}\xi \varepsilon Sus_{i}^{(IC}\\ \; & -\alpha env\left(T\right)\lambda \theta \frac{\left(\sigma_{Y}Sym_{Y}^{(Imm}+\sigma_{M}Sym_{M}^{(Imm}+\sigma_{S}Sym_{S}^{(Imm}\right)}{n}\xi \varepsilon Sus_{i}^{(IC}\\ \; & -\alpha env\left(T\right)\lambda \frac{\left(\sigma_{Y}Asym_{Y}^{(Imm}+\sigma_{M}Asym_{M}^{(Imm}+\sigma_{S}Asym_{S}^{(Imm}\right)}{n}\xi \varepsilon Sus_{i}^{(IC}\\ \; & -\alpha env\left(T\right)\lambda \theta \xi \frac{\left(\sigma_{Y}Sym_{Y}^{(IC}+\sigma_{M}Sym_{M}^{(IC}+\sigma_{S}Sym_{S}^{(IC}\right)}{n}\xi \varepsilon Sus_{i}^{(IC}\\ \; & +\rho Sym_{ICi}+\nu H_{ICi}+\beta_{i}-\frac{\beta_{i}}{n_{i}^{(IC}}Sus_{i}^{(IC} \end{array}$$ (1)

$$\begin{array}{cc} \frac{dE_{i}^{(IC}}{dt}=\; & +\;\alpha env\left(T\right)\lambda \theta \frac{\left(\sigma_{Y}Sym_{Y}+\sigma_{M}Sym_{M}+\sigma_{S}Sym_{S}\right)}{n}\xi \varepsilon Sus_{i}^{(IC}\\ \; & +\;\alpha env\left(T\right)\lambda \frac{\left(\sigma_{Y}Asym_{Y}+\sigma_{M}Asym_{M}+\sigma_{S}Asym_{S}\right)}{n}\xi \varepsilon Sus_{i}^{(IC}\\ \; & +\;\alpha env\left(T\right)\lambda \theta \frac{\left(\sigma_{Y}Sym_{Y}^{(Imm}+\sigma_{M}Sym_{M}^{(Imm}+\sigma_{S}Sym_{S}^{(Imm}\right)}{n}\xi \varepsilon Sus_{i}^{(IC}\\ \; & +\;\alpha env\left(T\right)\lambda \frac{\left(\sigma_{Y}Asym_{Y}^{(Imm}+\sigma_{M}Asym_{M}^{(Imm}+\sigma_{S}Asym_{S}^{(Imm}\right)}{n}\xi \varepsilon Sus_{i}^{(IC}\\ \; & +\;\alpha env\left(T\right)\lambda \theta \xi \frac{\left(\sigma_{Y}Sym_{Y}^{(IC}+\sigma_{M}Sym_{M}^{(IC}+\sigma_{S}Sym_{S}^{(IC}\right)}{n}\xi \varepsilon Sus_{i}^{(IC}\\ \; & -\;\tau E_{i}^{(IC} \end{array}$$ (2)

$$\frac{dSym_{i}^{(IC}}{dt}=\tau E_{i}^{(IC}-\varpi_{i}^{(IC}Sym_{i}^{(IC}-\rho Sym_{i}^{(IC}$$ (3)

$$\frac{dH_{i}^{(IC}}{dt}=\varpi_{i}^{(IC}Sym_{i}^{(IC}-\nu H_{i}^{(IC}-\delta_{i}^{(IC}H_{i}^{(IC}$$ (4)

$$\frac{dD_{i}^{(IC}}{dt}=\delta_{i}^{(IC}H_{i}^{(IC}$$ (5)

### Non-COVID-19 immunised immunocompetent population


$$\begin{array}{cc} \frac{dSus_{i}}{dt}= & -\alpha env\left(T\right)\lambda \theta \frac{\left(\sigma_{Y}Sym_{Y}+\sigma_{M}Sym_{M}+\sigma_{S}Sym_{S}\right)}{n}\sigma_{i}Sus_{i}\\ & -\alpha env\left(T\right)\lambda \frac{\left(\sigma_{Y}Asym_{Y}+\sigma_{M}Asym_{M}+\sigma_{S}Asym_{S}\right)}{n}\sigma_{i}Sus_{i}\\ & -\alpha env\left(T\right)\lambda \theta \frac{\left(\sigma_{Y}Sym_{Y}^{(Imm}+\sigma_{M}Sym_{M}^{(Imm}+\sigma_{S}Sym_{S}^{(Imm}\right)}{n}\sigma_{i}Sus_{i}\\ & -\alpha env\left(T\right)\lambda \frac{\left(\sigma_{Y}Asym_{Y}^{(Imm}+\sigma_{M}Asym_{M}^{(Imm}+\sigma_{S}ASym_{S}^{(Imm}\right)}{n}\sigma_{i}Sus_{i}\\ & -\alpha env\left(T\right)\lambda \theta \xi \frac{\left(\sigma_{Y}Sym_{Y}^{(IC}+\sigma_{M}Sym_{M}^{(IC}+\sigma_{S}Sym_{S}^{(IC}\right)}{n}\sigma_{i}Sus_{i}\\ & +\phi_{SusImm}Sus_{i}^{(Imm}-\kappa Sus_{i}-effi_{1sev}{vac}_{\text{1}}\left(Sus_{i}\right)-\beta_{i}+\frac{\beta_{i}}{n_{i}^{(IC}}Sus_{i}^{(IC} \end{array}$$ (6)

$$\begin{array}{cc} \frac{dE_{i}}{dt}= & +\alpha env\left(T\right)\lambda \theta \frac{\left(\sigma_{Y}Sym_{Y}+\sigma_{M}Sym_{M}+\sigma_{S}Sym_{S}\right)}{n}\sigma_{i}Sus_{i}\\ & +\alpha env\left(T\right)\lambda \frac{\left(\sigma_{Y}Asym_{Y}+\sigma_{M}Asym_{M}+\sigma_{S}Asym_{S}\right)}{n}\sigma_{i}Sus_{i}\\ & +\alpha env\left(T\right)\lambda \theta \frac{\left(\sigma_{Y}Sym_{Y}^{(Imm}+\sigma_{M}Sym_{M}^{(Imm}+\sigma_{S}Sym_{S}^{(Imm}\right)}{n}\sigma_{i}Sus_{i}\\ & +\alpha env\left(T\right)\lambda \frac{\left(\sigma_{Y}Asym_{Y}^{(Imm}+\sigma_{M}Asym_{M}^{(Imm}+\sigma_{S}Asym_{S}^{(Imm}\right)}{n}\sigma_{i}Sus_{i}\\ & +\alpha env\left(T\right)\lambda \theta \xi \frac{\left(\sigma_{Y}Sym_{Y}^{(IC}+\sigma_{M}Sym_{M}^{(IC}+\sigma_{S}Sym_{S}^{(IC}\right)}{n}\sigma_{i}Sus_{i}\\ & +\kappa Sus_{i}-\tau E_{i} \end{array}$$ (7)

$$\frac{dSym_{i}}{dt}=\tau (1-f_{Asym})E_{i}-\varpi_{i}Sym_{i}-\rho_{Sym}Sym_{i}$$ (8)

$$\frac{dAsym_{i}}{dt}=\tau f_{Asym}E_{i}-\rho_{Asym}Asym_{i}-effi_{1sev}{vac}_{\text{1}}\left(Asym_{i}\right)$$ (9)

$$\frac{dH_{i}}{dt}=\varpi_{i}Sym_{i}-\nu H_{i}-\delta_{i}H_{i}$$ (10)

$$\frac{dRInf_{i}}{dt}=\;\rho_{Sym}Sym_{i}+\rho_{Asym}Asym_{i}+\nu H_{i}\;\;\;\;-\phi_{RInf}RInf_{i}-effi_{1sev}{vac}_{\text{1}}\left(RInf_{i}\right)$$ (11)

$$\frac{dD_{i}}{dt}=\delta_{i}H_{i},$$ (12)

### COVID-19 immunised immunocompetent population


$$\begin{array}{cc} \frac{dSus_{i}^{(Imm}}{dt}= & -\alpha env\left(T\right)\lambda \theta \frac{\left(\sigma_{Y}Sym_{Y}+\sigma_{M}Sym_{M}+\sigma_{S}Sym_{S}\right)}{n}\sigma_{i}Sus_{i}^{(Imm}\\ & -\alpha env\left(T\right)\lambda \frac{\left(\sigma_{Y}Asym_{Y}+\sigma_{M}Asym_{M}+\sigma_{S}Asym_{S}\right)}{n}\sigma_{i}Sus_{i}^{(Imm}\\ & -\alpha env\left(T\right)\lambda \theta \frac{\left(\sigma_{Y}Sym_{Y}^{(Imm}+\sigma_{M}Sym_{M}^{(Imm}+\sigma_{S}Sym_{S}^{(Imm}\right)}{n}\sigma_{i}Sus_{i}^{(Imm}\\ & -\alpha env\left(T\right)\lambda \frac{\left(\sigma_{Y}Asym_{Y}^{(Imm}+\sigma_{M}Asym_{M}^{(Imm}+\sigma_{S}Asym_{S}^{(Imm}\right)}{n}\sigma_{i}Sus_{i}^{(Imm}\\ & -\alpha env\left(T\right)\lambda \theta \xi \frac{\left(Sym_{Y}^{(IC}+\sigma_{M}Sym_{M}^{(IC}+\sigma_{S}Sym_{S}^{(IC}\right)}{n}\sigma_{i}Sus_{i}^{(Imm}\\ & +\phi_{RInf}RInf_{i}+\phi_{RMix}RMix_{i}\\ & +\left(effi_{1sev}-\frac{effi_{1mild}}{effi_{1sev}}\right)\left({vac}_{\text{1}}\left(Sus_{i})+{vac}_{\text{1}}(Asym_{i})+{vac}_{\text{1}}(RInfc_{i}\right)\right)\\ & \text{ -}\kappa Sus_{i}^{(Imm}-\phi_{SusImm}Sus_{i}^{(Imm}-effi_{boost}boost\left(Sus_{i}^{(Imm}\right) \end{array}$$ (13)

$$\begin{array}{cc} \frac{dE_{i}^{Imm}}{dt}= & +\alpha env\left(T\right)\lambda \theta \frac{\left(\sigma_{Y}Sym_{Y}+\sigma_{M}Sym_{M}+\sigma_{S}Sym_{S}\right)}{n}\sigma_{i}Sus_{i}^{(Imm}\\ & +\alpha env\left(T\right)\lambda \frac{\left(\sigma_{Y}Asym_{Y}+\sigma_{M}Asym_{M}+\sigma_{S}Asym_{S}\right)}{n}\sigma_{i}Sus_{i}^{(Imm}\\ & +\alpha env\left(T\right)\lambda \theta \frac{\left(\sigma_{Y}Sym_{Y}^{(Imm}+\sigma_{M}Sym_{M}^{(Imm}+\sigma_{S}Sym_{S}^{(Imm}\right)}{n}\sigma_{i}Sus_{i}^{(Imm}\\ & +\alpha env\left(T\right)\lambda \frac{\left(\sigma_{Y}Asym_{Y}^{(Imm}+\sigma_{M}Asym_{M}^{(Imm}+\sigma_{S}Asym_{S}^{(Imm}\right)}{n}\sigma_{i}Sus_{i}^{(Imm}\\ & +\alpha env\left(T\right)\lambda \theta \xi \frac{\left(Sym_{Y}^{(IC}+\sigma_{M}Sym_{M}^{(IC}+\sigma_{S}Sym_{S}^{(IC}\right)}{n}\sigma_{i}Sus_{i}^{(Imm}\\ & +\kappa Sus_{i}^{Imm}-\tau E_{i}^{Imm} \end{array}$$ (14)

$$\frac{dSym_{i}^{(Imm}}{dt}=\tau (1-f_{Asym})E_{i}^{(Imm}-\rho_{Asym}Sym_{i}^{(Imm}$$ (15)

$$\frac{dAsym_{i}^{(Imm}}{dt}=\tau f_{Asym}E_{i}^{(Imm}-\rho_{Asym}Asym_{i}^{(Imm}-effi_{boost}boost\left(Asym_{i}^{(Imm}\right)$$ (16)

$$\begin{array}{cc} \frac{dRMix_{i}}{dt}= & +\rho_{Asym}Sym_{i}^{(Imm}+\rho_{Asym}Asym_{i}^{(Imm}-\phi_{RMix}RMix_{i}\\ & +\frac{effi_{1mild}}{effi_{1sev}}\left({vac}_{\text{1}}\left(Sus_{i})+{vac}_{\text{1}}(Asym_{i})+{vac}_{\text{1}}(RInfc_{i}\right)\right)\\ & +effi_{boost}\left(boost\left(Sus_{i}^{(Imm})+boost(Asym_{i}^{(Imm}\right)\right) \end{array}$$ (17)

Two further equations describe the number of new infection cases and hospital admissions per unit time for SIC subjects:


$$\frac{dNewC_{i}^{(IC}}{dt}=\tau E_{i}^{(IC}$$ (18)

$$\frac{dAdm_{i}^{IC}}{dt}=\varpi_{i}^{(IC}Sym_{i}^{(IC},$$ (19)

as well as for the immunocompetent population:


$$\frac{dNewC_{i}}{dt}=\tau E_{i}+\tau E_{i}^{(Imm}$$ (20)

$$\frac{dAdm_{i}}{dt}=\varpi_{i}Sym_{i}$$ (21)

where i={Y,M,S} indicates the age group, and *t* denotes time (days). Sta*t*e variables are defined above, and parameter values are included in Table 1. Initial values used to solve the model are provided in Supplementary Table S1 in S1 File.

To identify parameter values, we used quantitative information on COVID-19 cases made available by the UK government and other publicly available sources (Table 1, Fig 2, Supplementary Table S1, Supplementary Figures S1B-C in S1 File) from August 1^st^, 2020, to December 31^st^, 2022. Model simulations were performed using MATLAB software (version R2024b).

MATLAB (version R2024b) model codes and data are available in Supplementary Material and deposited in BioModels (MODEL2502220001) database [46]. Data used in this paper are publicly available and all sources are cited.

### Model sensitivity analysis

The relative (dimensionless) sensitivity value, *S*_*ij*_*,* of the model variable *X*_*i*_ to the parameter *p*_*j*_, was defined through the partial derivative $S_{ij}=\frac{dX_{i}}{dp_{j}}\frac{p_{j}}{X_{i}}$ and computed using the *ode* function available in MATLAB 2024b. Thus, *S*_*ij*_ quantifies the percentage change in the variable *X*_*i*_ per percentage change in the parameter *p*_*j*_. Values of *S* near zero indicate that the output is insensitive to that parameter, whereas values greater than one indicate that changes in the parameter value lead to proportionally larger changes in the output variable. Positive (negative) values indicate that the output variable increases (decreases) when the parameter increases.

We analysed the sensitivity of 54 model state variables to 13 parameters. We estimated the total effect of each parameter as the sum of absolute sensitivity values across all state variables, as well as, separately within the immunocompetent and SIC populations to identify which population was most affected by each parameter (Supplementary Table S2 in S1 File). To avoid redundancies, we did not evaluate the effects of multiplicative rate modifiers associated with discrete events (for example, variant emergence or implementation of protective strategies). We likewise did not examine sensitivity to the timing of such events.

## Supporting information

S1 File Supporting Information Figures and Tables (WORD file). (DOCX)

S2 File MATLAB model code (ZIP file). (ZIP)

## References

[1] World Health Organization. WHO timeline - COVID-19. World Health Organization. https://www.who.int/news/item/27-04-2020-who-timeline---covid-19 2023 October 1.

[2] Flahault A, Calmy A, Costagliola D, Drapkina O, Eckerle I, Larson HJ, et al. No time for complacency on COVID-19 in Europe. Lancet. 2023;401(10392):1909–12. doi: 10.1016/S0140-6736(23)01012-7 37230103 PMC10202416

[3] NHS. Landmark moment as first NHS patient receives COVID-19 vaccination. https://www.england.nhs.uk/2020/12/landmark-moment-as-first-nhs-patient-receives-covid-19-vaccination/

[4] Mounier-Jack S, Paterson P, Bell S, Letley L, Kasstan B, Chantler T. Covid-19 vaccine roll-out in England: A qualitative evaluation. PLoS One. 2023;18(6):e0286529. doi: 10.1371/journal.pone.0286529 37267295 PMC10237459

[5] NHS E. COVID-19 weekly announced vaccinations: Week ending Sunday 23rd January 2022. 2022. https://www.england.nhs.uk/statistics/wp-content/uploads/sites/2/2022/01/COVID-19-weekly-announced-vaccinations-27-January-2022.pdf

[6] Turtle L, Thorpe M, Drake TM, Swets M, Palmieri C, Russell CD, et al. Outcome of COVID-19 in hospitalised immunocompromised patients: An analysis of the WHO ISARIC CCP-UK prospective cohort study. PLoS Med. 2023;20(1):e1004086. doi: 10.1371/journal.pmed.1004086 36719907 PMC9928075

[7] Singson JRC, Kirley PD, Pham H, Rothrock G, Armistead I, Meek J, et al. Factors Associated with Severe Outcomes Among Immunocompromised Adults Hospitalized for COVID-19 - COVID-NET, 10 States, March 2020-February 2022. MMWR Morb Mortal Wkly Rep. 2022;71(27):878–84. doi: 10.15585/mmwr.mm7127a3 35797216 PMC9290380

[8] Nevejan L, Ombelet S, Laenen L, Keyaerts E, Demuyser T, Seyler L, et al. Severity of COVID-19 among Hospitalized Patients: Omicron Remains a Severe Threat for Immunocompromised Hosts. Viruses. 2022;14(12):2736. doi: 10.3390/v14122736 36560741 PMC9783877

[9] Evans RA, Dube S, Lu Y, Yates M, Arnetorp S, Barnes E, et al. Impact of COVID-19 on immunocompromised populations during the Omicron era: insights from the observational population-based INFORM study. Lancet Reg Health Eur. 2023;35:100747. doi: 10.1016/j.lanepe.2023.100747 38115964 PMC10730312

[10] Shoham S, Batista C, Ben Amor Y, Ergonul O, Hassanain M, Hotez P, et al. Vaccines and therapeutics for immunocompromised patients with COVID-19. EClinicalMedicine. 2023;59:101965. doi: 10.1016/j.eclinm.2023.101965 37070102 PMC10091856

[11] Metcalf CJE, Morris DH, Park SW. Mathematical models to guide pandemic response. Science. 2020;369(6502):368–9. doi: 10.1126/science.abd1668 32703861

[12] Bjørnstad ON, Shea K, Krzywinski M, Altman N. The SEIRS model for infectious disease dynamics. Nat Methods. 2020;17(6):557–8. doi: 10.1038/s41592-020-0856-2 32499633

[13] Giordano G, Blanchini F, Bruno R, Colaneri P, Di Filippo A, Di Matteo A, et al. Modelling the COVID-19 epidemic and implementation of population-wide interventions in Italy. Nat Med. 2020;26(6):855–60. doi: 10.1038/s41591-020-0883-7 32322102 PMC7175834

[14] van Wees JD, Osinga S, van der Kuip M, Tanck M, Hanegraaf M, Pluymaekers M. Forecasting hospitalization and ICU rates of the COVID-19 outbreak: an efficient SEIR model. Bull World Health Organ.

[15] Blyuss KB, Kyrychko YN. Efects of latency and age structure on the dynamics and containment of COVID-19. 2020. https://www.medrxiv.org/content/10.1101/2020.04.25.20079848v1 10.1016/j.jtbi.2021.110587 PMC8143904 33450286

[16] Kyrychko YN, Blyuss KB, Brovchenko I. Mathematical modelling of the dynamics and containment of COVID-19 in Ukraine. Sci Rep. 2020;10(1):19662. doi: 10.1038/s41598-020-76710-1 33184338 PMC7665000

[17] Odagaki T. New compartment model for COVID-19. Sci Rep. 2023;13(1):5409. doi: 10.1038/s41598-023-32159-6 37012332 PMC10068699

[18] Ambalarajan V, Mallela AR, Sivakumar V, Dhandapani PB, Leiva V, Martin-Barreiro C, et al. A six-compartment model for COVID-19 with transmission dynamics and public health strategies. Sci Rep. 2024;14(1):22226. doi: 10.1038/s41598-024-72487-9 39333156 PMC11436938

[19] Mehrabi Nejad M-M, Moosaie F, Dehghanbanadaki H, Haji Ghadery A, Shabani M, Tabary M, et al. Immunogenicity of COVID-19 mRNA vaccines in immunocompromised patients: a systematic review and meta-analysis. Eur J Med Res. 2022;27(1):23. doi: 10.1186/s40001-022-00648-5 35151362 PMC8840778

[20] Bahremand T, Yao JA, Mill C, Piszczek J, Grant JM, Smolina K. COVID-19 hospitalisations in immunocompromised individuals in the Omicron era: a population-based observational study using surveillance data in British Columbia, Canada. Lancet Reg Health Am. 2023;20:100461. doi: 10.1016/j.lana.2023.100461 36890850 PMC9987330

[21] DeWolf S, Laracy JC, Perales M-A, Kamboj M, van den Brink MRM, Vardhana S. SARS-CoV-2 in immunocompromised individuals. Immunity. 2022;55(10):1779–98. doi: 10.1016/j.immuni.2022.09.006 36182669 PMC9468314

[22] UK Office for National Statistics. Principal projection - UK population in age groups. https://www.ons.gov.uk/peoplepopulationandcommunity/populationandmigration/populationprojections/datasets/tablea21principalprojectionukpopulationinagegroups 2023 October 1.

[23] The Official Website of the City of New York. Report of Coronavirus Disease 2019 (COVID-19): Deaths Among Confirmed Cases. 2020. https://www1.nyc.gov/assets/doh/downloads/pdf/imm/covid-19-daily-data-summary-deaths-05132020-1.pdf

[24] Docherty AB, Harrison EM, Green CA, Hardwick HE, Pius R, Norman L, et al. Features of 20 133 UK patients in hospital with covid-19 using the ISARIC WHO Clinical Characterisation Protocol: prospective observational cohort study. BMJ. 2020;369:m1985. doi: 10.1136/bmj.m1985 32444460 PMC7243036

[25] Centers for Disease Control and Prevention. Report of Hospitalization Rates and Characteristics of Patients Hospitalized with Laboratory-Confirmed Coronavirus Disease 2019. https://www.cdc.gov/mmwr/volumes/69/wr/mm6915e3.htm

[26] Wallinga J, Teunis P, Kretzschmar M. Using data on social contacts to estimate age-specific transmission parameters for respiratory-spread infectious agents. Am J Epidemiol. 2006;164(10):936–44. doi: 10.1093/aje/kwj317 16968863

[27] McCrone JT, Hill V, Bajaj S, Pena RE, Lambert BC, Inward R, et al. Context-specific emergence and growth of the SARS-CoV-2 Delta variant. Nature. 2022;610(7930):154–60. doi: 10.1038/s41586-022-05200-3 35952712 PMC9534748

[28] Nyberg T, Ferguson NM, Nash SG, Webster HH, Flaxman S, Andrews N, et al. Comparative analysis of the risks of hospitalisation and death associated with SARS-CoV-2 omicron (B.1.1.529) and delta (B.1.617.2) variants in England: a cohort study. Lancet. 2022;399(10332):1303–12. doi: 10.1016/S0140-6736(22)00462-7 35305296 PMC8926413

[29] Sajadi MM, Habibzadeh P, Vintzileos A, Shokouhi S, Miralles-Wilhelm F, Amoroso A. Temperature, Humidity, and Latitude Analysis to Estimate Potential Spread and Seasonality of Coronavirus Disease 2019 (COVID-19). JAMA Netw Open. 2020;3(6):e2011834. doi: 10.1001/jamanetworkopen.2020.11834 32525550 PMC7290414

[30] Merow C, Urban MC. Seasonality and uncertainty in global COVID-19 growth rates. Proc Natl Acad Sci U S A. 2020;117(44):27456–64. doi: 10.1073/pnas.2008590117 33051302 PMC7959558

[31] Tande AJ, Binnicker MJ, Ting HH, Del Rio C, Jalil L, Brawner M, et al. SARS-CoV-2 Testing Before International Airline Travel, December 2020 to May 2021. Mayo Clin Proc. 2021;96(11):2856–60. doi: 10.1016/j.mayocp.2021.08.019 34736612 PMC8410576

[32] NHS, COVID-19 Vaccinations. https://www.england.nhs.uk/statistics/statistical-work-areas/covid-19-vaccinations/

[33] Polack FP, Thomas SJ, Kitchin N, Absalon J, Gurtman A, Lockhart S. Safety and Efficacy of the BNT162b2 mRNA Covid-19 Vaccine. N Engl J Med. 2020. 10.1056/NEJMoa2034577 PMC7745181 33301246

[34] Jackson LA, Anderson EJ, Rouphael NG, Roberts PC, Makhene M, Coler RN. An mRNA Vaccine against SARS-CoV-2 - Preliminary Report. N Engl J Med. 2020;383(20):1920–31. 32663912 10.1056/NEJMoa2022483 PMC7377258

[35] Knoll MD, Wonodi C. Oxford-AstraZeneca COVID-19 vaccine efficacy. Lancet. 2020. 10.1016/S0140-6736(20)32623-4 PMC7832220 33306990

[36] Sadoff J, Gray G, Vandebosch A, Cárdenas V, Shukarev G, Grinsztejn B, et al. Safety and Efficacy of Single-Dose Ad26.COV2.S Vaccine against Covid-19. N Engl J Med. 2021;384(23):2187–201. doi: 10.1056/NEJMoa2101544 33882225 PMC8220996

[37] Feikin DR, Higdon MM, Abu-Raddad LJ, Andrews N, Araos R, Goldberg Y, et al. Duration of effectiveness of vaccines against SARS-CoV-2 infection and COVID-19 disease: results of a systematic review and meta-regression. Lancet. 2022;399(10328):924–44. doi: 10.1016/S0140-6736(22)00152-0 35202601 PMC8863502

[38] Chemaitelly H, Tang P, Hasan MR, AlMukdad S, Yassine HM, Benslimane FM, et al. Waning of BNT162b2 Vaccine Protection against SARS-CoV-2 Infection in Qatar. N Engl J Med. 2021;385(24):e83. doi: 10.1056/NEJMoa2114114 34614327 PMC8522799

[39] Mizrahi B, Lotan R, Kalkstein N, Peretz A, Perez G, Ben-Tov A, et al. Correlation of SARS-CoV-2-breakthrough infections to time-from-vaccine. Nat Commun. 2021;12(1):6379. doi: 10.1038/s41467-021-26672-3 34737312 PMC8569006

[40] Goldberg Y, Mandel M, Bar-On YM, Bodenheimer O, Freedman L, Haas EJ, et al. Waning Immunity after the BNT162b2 Vaccine in Israel. N Engl J Med. 2021;385(24):e85. doi: 10.1056/NEJMoa2114228 34706170 PMC8609604

[41] Medić S, Anastassopoulou C, Lozanov-Crvenković Z, Vuković V, Dragnić N, Petrović V, et al. Risk and severity of SARS-CoV-2 reinfections during 2020-2022 in Vojvodina, Serbia: A population-level observational study. Lancet Reg Health Eur. 2022;20:100453. doi: 10.1016/j.lanepe.2022.100453 35791336 PMC9246704

[42] The official UK Government website for data and insights on Coronavirus (COVID-19). https://coronavirus.data.gov.uk/details/download 2023 October 1.

[43] Belsky JA, Tullius BP, Lamb MG, Sayegh R, Stanek JR, Auletta JJ. COVID-19 in immunocompromised patients: A systematic review of cancer, hematopoietic cell and solid organ transplant patients. J Infect. 2021;82(3):329–38. doi: 10.1016/j.jinf.2021.01.022 33549624 PMC7859698

[44] Watson OJ, Barnsley G, Toor J, Hogan AB, Winskill P, Ghani AC. Global impact of the first year of COVID-19 vaccination: a mathematical modelling study. Lancet Infect Dis. 2022;22(9):1293–302. doi: 10.1016/S1473-3099(22)00320-6 35753318 PMC9225255

[45] Suarez Castillo M, Khaoua H, Courtejoie N. Vaccine-induced and naturally-acquired protection against Omicron and Delta symptomatic infection and severe COVID-19 outcomes, France, December 2021 to January 2022. Euro Surveill. 2022;27(16):2200250. doi: 10.2807/1560-7917.ES.2022.27.16.2200250 35451363 PMC9027152

[46] Malik-Sheriff RS, Glont M, Nguyen TVN, Tiwari K, Roberts MG, Xavier A, et al. BioModels-15 years of sharing computational models in life science. Nucleic Acids Res. 2020;48(D1):D407–15. doi: 10.1093/nar/gkz1055 31701150 PMC7145643

[47] UK Office for National Statistics. Report of Coronavirus (COVID-19) weekly insights: latest health indicators in England. 2020. https://www.ons.gov.uk/peoplepopulationandcommunity/healthandsocialcare/conditionsanddiseases/articles/coronaviruscovid19weeklyinsights/latesthealthindicatorsinengland11december2020

[48] UK Meteorological Office. The UK Meteorological Office. https://www.metoffice.gov.uk/research/climate/maps-and-data/historic-station-data 2023 October 1.

[49] Lavezzo E, Franchin E, Ciavarella C, Cuomo-Dannenburg G, Barzon L, Del Vecchio C, et al. Suppression of a SARS-CoV-2 outbreak in the Italian municipality of Vo’. Nature. 2020;584(7821):425–9. doi: 10.1038/s41586-020-2488-1 32604404 PMC7618354

[50] Takahashi K, Ishikane M, Ujiie M, Iwamoto N, Okumura N, Sato T, et al. Duration of Infectious Virus Shedding by SARS-CoV-2 Omicron Variant-Infected Vaccinees. Emerg Infect Dis. 2022;28(5):998–1001. doi: 10.3201/eid2805.220197 35290176 PMC9045443

[51] European Centre for Disease Prevention and Control. Report of Novel coronavirus (SARS-CoV-2) Discharge criteria for confirmed COVID-19 cases -When is it safe to discharge COVID-19 cases from the hospital or end home isolation? https://www.ecdc.europa.eu/sites/default/files/documents/COVID-19-Discharge-criteria.pdf

[52] Zou L, Ruan F, Huang M, Liang L, Huang H, Hong Z, et al. SARS-CoV-2 Viral Load in Upper Respiratory Specimens of Infected Patients. N Engl J Med. 2020;382(12):1177–9. doi: 10.1056/NEJMc2001737 32074444 PMC7121626

[53] World Health Organization. Report of COVID-19 weekly surveillance. 2021. https://www.euro.who.int/__data/assets/pdf_file/0003/445089/Week-21-COVID-19-surveillance-report-eng.pdf?ua=1

[54] UK Office for National Statistics analysis on people with long-term health conditions, UK: January to December 2019. https://www.ons.gov.uk/peoplepopulationandcommunity/healthandsocialcare/conditionsanddiseases/adhocs/11478peoplewithlongtermhealthconditionsukjanuarytodecember2019

