# Supplementary material for: A dynamic model of COVID-19 infection quantifies the impact of preventive interventions on the infection of severely immunocompromised subjects in the United Kingdom
Source: PLoS One. 2026 Feb 23;21(2):e0341331. doi: 10.1371/journal.pone.0341331 (PMC12928435; doi:10.1371/journal.pone.0341331)
Supplement: S1 File — (DOCX) [file pone.0341331.s001.docx]

# Supporting Information: Figures and Tables

**A dynamic model of COVID-19 infection quantifies the impact of preventive interventions on the infection of severely immunocompromised subjects in the United Kingdom**

Carmen Pin^1*^, Sylvia Taylor^2^, Catia Ferreira^3^, Sofie Arnetorp^4^, Holly Kimko^5^

## **Supplementary Figures**


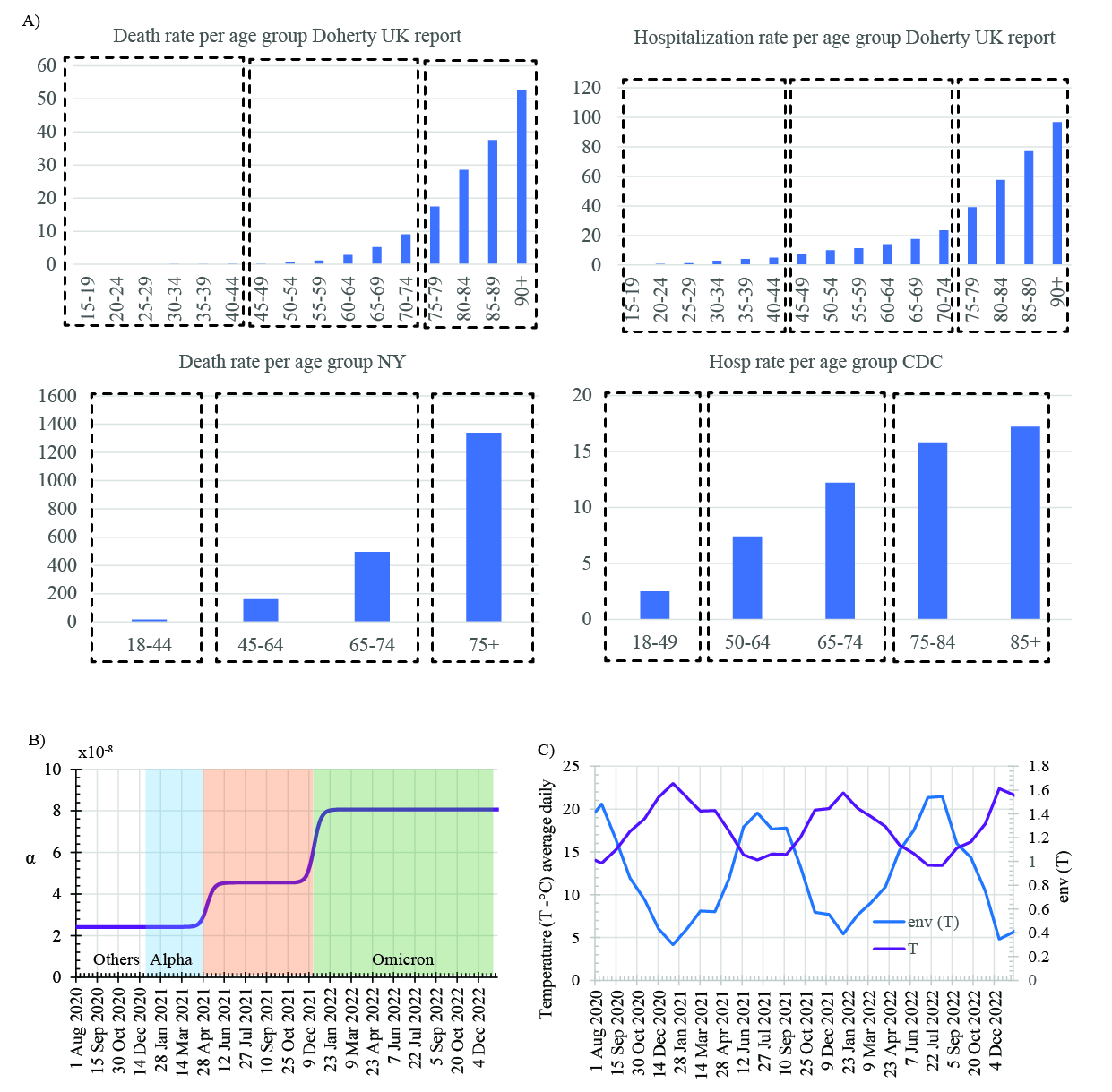


**Supplementary Figure S1.** A) Selection criterium of three age groups. Age groups were chosen to minimize within-group heterogeneity in hospitalization and mortality rates (per 100,000 subjects in the age group) reported across multiple sources [22-25], while maximizing differences between groups. Discontinuous lines depict the published hospitalization and death rates by age group [22-25] within each age group, consistent with this criterion. Where applicable, rates were calculated as the number of reported patients divided by the corresponding age-group population and multiplied by 100,000. Rates were used to assess age impact within each dataset. Variation in reported rates between datasets reflects that counts were gathered over differing observation periods and timing across sources; B) Representation of the function, *α*, quantifying changes in COVID-19 transmission rate according to the reported timeline of the emergence of the alpha (B.1.1.7), delta (B.1.617.2) and omicron (B.1.1.529) variants in the UK from August 1^st^, 2020, to December 31st, 2022 [27, 28]; C) Representation of the function, env(*T*), that quantifies changes in COVID-19 transmission rate responding to changes in temperature, *T*. Temperature values are the monthly averages reported for Heathrow climate station by the UK Met Office from August 1^st^, 2020, to December 31st, 2022 [48].


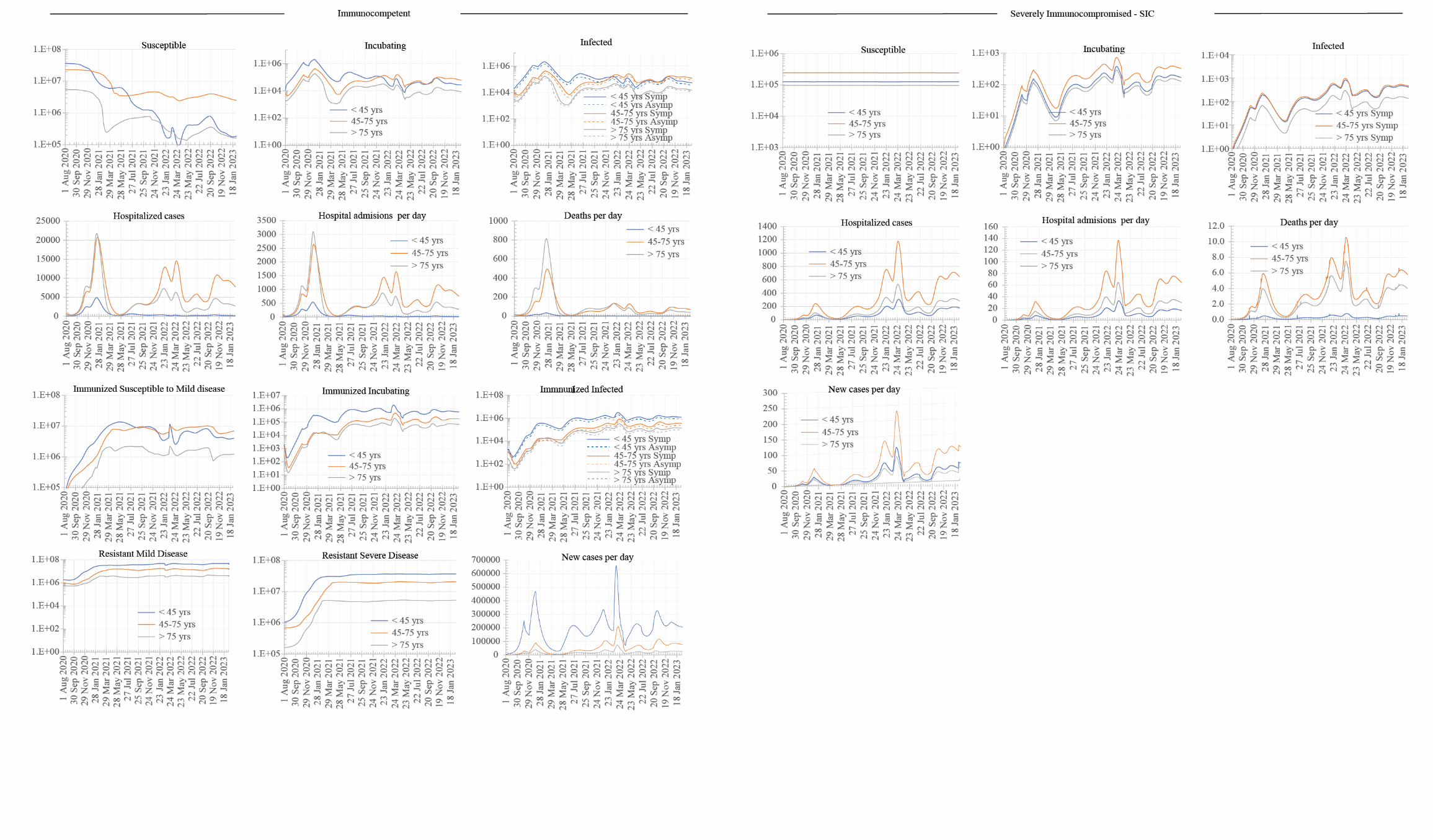


**Supplementary Figure S2.** Predicted COVID-19 infection dynamics in each age group from August 1^st^, 2020, to December 31st, 2022, in the UK. The model considers two national lockdown periods, isolation during symptomatic disease, reduced transmission during school holiday, infection seasonality, international air travelling restrictions, emergence of new COVID-19 variants and age differences in social activity and overall health condition. The administration of the COVID-19 vaccine was implemented using the actual vaccination deployment rates published by NHS England during 2021 and 2022 for each age group, which include up to four doses for subjects aged 45 and above [32`] (Figure 1B)

**
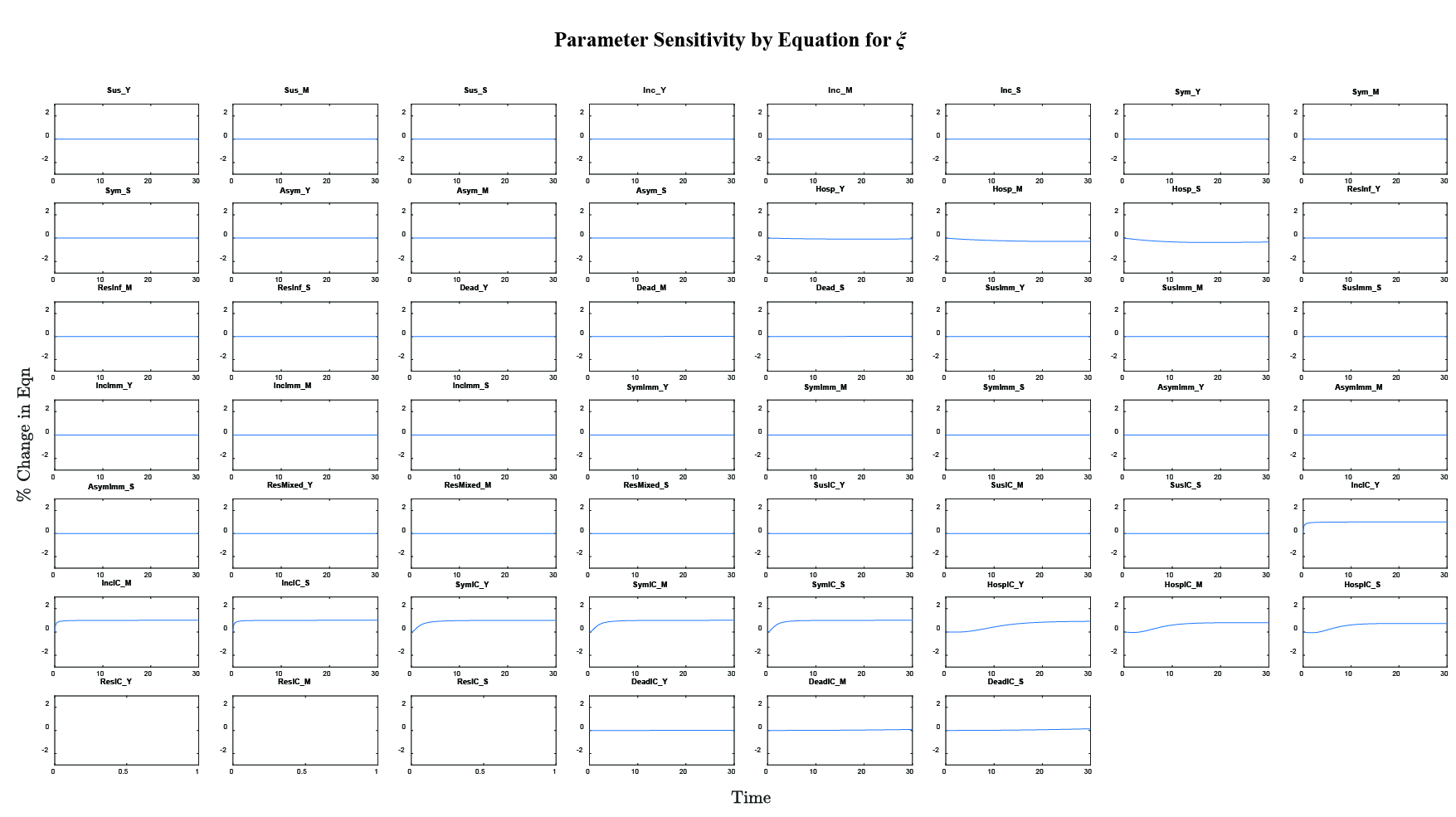
**

**Supplementary Figure S3.** Sensitivity values of parameter *ξ* which captures modification of infection transmission of SIC individuals.

## **Table S1**

**Supplementary Table S1. Initial values of the model state variables**

| Variable* | Value | Unit |
| --- | --- | --- |
| *Sus_Y_* (0)*, Sus_M_* (0)*, Sus_S_* (0) | 35947058, 22956503, 5432791 | subjects |
| *E_Y_* (0)*, E_M_* (0), *E_S_* (0) | 11270, 7197, 1703 | subjects |
| *Sym_Y_* (0), *Sym_M_* (0), *Sym_S_* (0) | 21639, 13819, 3270 | subjects |
| *Asym_Y_* (0), *Asym_M_* (0), *Asym_S_* (0) | 14426, 9213, 2180 | subjects |
| *H_Y_* (0), *H_M_* (0), *H_S_* (0) | 129, 606, 555 | subjects |
| *RInf_Y_* (0), *RInf_M_* (0), *RInf_S_* (0) | 1025732, 655053, 155022 | subjects |
| *D_Y_* (0), *D_M_ (0)*, *D_S_* (0) | 2868, 25816, 28684 | subjects |
| *Sus^(Imm^_Y_* (0)*, Sus^(Imm^_M_* (0)*, Sus^(Imm^_S_* (0) | 12308, 7860, 1860 | subjects |
| *E^(Imm^_Y_* (0)*, E^(Imm^_M_* (0), *E ^(Imm^_S_* (0) | 2051, 1310, 310 | subjects |
| *Sym^(Imm^_Y_* (0), *Sym^(Imm^_M_* (0), *Sym^(Imm^_S_* (0) | 2051, 1310, 310 | subjects |
| *Asym^(Imm^_Y_* (0), *Asym^(Imm^_M_* (0), *Asym^(Imm^_S_* (0) | 2051, 1310, 310 | subjects |
| *RMix_Y_* (0), *RMix_M_* (0), *RMix_S_* (0) | 2051, 1310, 310 | subjects |
| *Sus^(IC^_Y_* (0)*, Sus^(IC^_M_* (0)*, Sus^(IC^_S_* (0) |  | subjects |
| *E^(IC^_Y_* (0)*, E^(IC^_M_* (0), *E^(IC^_S_* (0) | 126469, 243963, 96891 | subjects |
| *Sym^(IC^_Y_* (0), *Sym^(IC^_M_* (0), *Sym^(IC^_S_* (0) | 0, 0, 0 | subjects |
| *Asym^(IC^_Y_* (0), *Asym^(IC^_M_* (0), *Asym^(IC^_S_* (0) | 0, 0, 0 | subjects |
| *H^(IC^_Y_* (0), *H^(IC^_M_* (0), *H^(IC^_S_* (0) | 0, 0, 0 | subjects |
| *D^(IC^_Y_* (0), *D^(IC^_M_ (0)*, *D^(IC^_S_* (0) | 109, 211, 83 | subjects |
|  |  |  |
| *NewC_Y_* (0), *NewC_M_* (0), *NewC_S_* (0), | 2996, 507, 1106 | subjects |
| *Adm_Y_* (0), *Adm_M_* (0), *Adm_S_* (0), | 21, 3, 7 | subjects |
| *NewC^(IC^_Y_* (0), *NewC^(IC^_M_* (0), *NewC^(IC^_S_* (0), | 18, 86, 79 | subjects |
| *Adm^(IC^_Y_* (0), *Adm^(IC^_M_* (0), *Adm^(IC^_S_* (0), | 0, 0, 0 | subjects |

*Day 0 is August 1^st^, 2020

## **Table S2**

**Supplementary Table S2. Sensitivity of the model state variables to model parameter values**

|  |  | Parameter | | | | | | | | | |
| --- | --- | --- | --- | --- | --- | --- | --- | --- | --- | --- | --- |
|  |  | *α* | *τ* | *ω* | *ρ_Sym_* | *ρ_Asym_* | *ν* | *f_Asym_* | *φ* | *δ* | *ξ* |
| Variable | *Sus_Y_* | -0.99 | -0.04 | 0 | 0 | 0 | 0 | -0.01 | 0 | -0.99 | 0 |
|  | *Sus_M_* | -0.99 | 0 | 0 | 0 | 0 | 0 | 0 | 0 | -0.99 | 0 |
|  | *Sus_S_* | -0.99 | -0.01 | 0 | 0 | 0 | 0 | 0 | 0 | -0.99 | 0 |
|  | *E_Y_* | 0 | -0.12 | 0 | -0.01 | -0.17 | 0 | 1.23 | 0 | 0 | 0 |
|  | *E_M_* | 0 | -0.2 | 0 | -0.01 | -0.03 | 0 | 1.09 | 0 | 0 | 0 |
|  | *E_S_* | 0 | -0.14 | 0 | -0.01 | -0.07 | 0 | 1.17 | 0 | 0 | 0 |
|  | *Sym_Y_* | 0 | 0.79 | -0.01 | -0.57 | -0.08 | 0 | 0.43 | 0 | 0 | 0 |
|  | *Sym_M_* | 0 | 0.67 | -0.34 | -0.69 | -0.01 | 0 | 0.34 | 0 | 0 | 0 |
|  | *Sym_S_* | 0 | 0.75 | -0.63 | -0.58 | -0.03 | 0 | 0.42 | 0 | 0 | 0 |
|  | *Asym_Y_* | 0 | 0.79 | 0 | -0.01 | -1.23 | 0 | 1.58 | 0 | 0 | 0 |
|  | *Asym_M_* | 0 | 0.66 | 0 | 0 | -1.1 | 0 | 1.29 | 0 | 0 | 0 |
|  | *Asym_S_* | 0 | 0.73 | 0 | -0.01 | -1.14 | 0 | 1.44 | 0 | 0 | 0 |
|  | *H_Y_* | 0 | 0.64 | 0.87 | -0.39 | -0.03 | -0.68 | 0.3 | 0 | -0.11 | -0.06 |
|  | *H_M_* | 0 | 0.56 | 0.68 | -0.5 | 0 | -0.67 | 0.21 | 0 | -0.24 | -0.2 |
|  | *H_S_* | 0 | 0.63 | 0.43 | -0.43 | -0.02 | -0.6 | 0.3 | 0 | -0.31 | -0.28 |
|  | *RInf_Y_* | 0 | 0.27 | 0 | 0.02 | 0 | 0 | 0.13 | 0.13 | 0 | 0 |
|  | *RInf_M_* | 0 | 0.12 | 0 | 0.01 | 0 | 0 | 0.02 | 0.15 | 0 | 0 |
|  | *RInf_S_* | 0 | 0.18 | 0 | 0.01 | 0 | 0 | 0.05 | 0.14 | 0 | 0 |
|  | *D_Y_* | 0 | 0.01 | 0.02 | 0 | 0 | 0 | 0 | 0 | 0.08 | 0 |
|  | *D_M_* | 0 | 0.01 | 0.03 | 0 | 0 | 0 | 0 | 0 | 0.09 | 0 |
|  | *D_S_* | 0 | 0.02 | 0.01 | 0 | 0 | 0 | 0 | 0 | 0.07 | 0 |
|  | *Sus^(Imm^_Y_* | -0.99 | 0.09 | 0 | 0.01 | 0 | 0 | 0.02 | -0.78 | -1 | 0 |
|  | *Sus^(Imm^_M_* | -1 | 0.02 | 0 | 0 | 0 | 0 | 0 | -0.77 | -1 | 0 |
|  | *Sus^(Imm^_S_* | -0.99 | 0.04 | 0 | 0 | 0 | 0 | 0 | -0.78 | -1 | 0 |
|  | *E^(Imm^_Y_* | 0 | -0.32 | 0 | 0 | -0.02 | 0 | 1.13 | -0.46 | 0 | 0 |
|  | *E^(Imm^_M_* | 0 | -0.78 | 0 | 0 | 0 | 0 | 0.92 | -0.37 | 0 | 0 |
|  | *E^(Imm^_S_* | 0 | -0.58 | 0 | 0 | -0.01 | 0 | 1.03 | -0.41 | 0 | 0 |
|  | *Sym^(Imm^_Y_* | 0 | 0.66 | 0 | 0 | -1.11 | 0 | 0.31 | -0.25 | 0 | 0 |
|  | *Sym^(Imm^_M_* | 0 | 0.44 | 0 | 0 | -1.09 | 0 | 0.1 | -0.15 | 0 | 0 |
|  | *Sym^(Imm^_S_* | 0 | 0.53 | 0 | 0 | -1.09 | 0 | 0.2 | -0.19 | 0 | 0 |
|  | *Asym^(Imm^_Y_* | 0 | 0.65 | 0 | 0 | -1.12 | 0 | 1.12 | -0.24 | 0 | 0 |
|  | *Asym^(Imm^_M_* | 0 | 0.44 | 0 | 0 | -1.1 | 0 | 0.81 | -0.14 | 0 | 0 |
|  | *Asym^(Imm^_S_* | 0 | 0.52 | 0 | 0 | -1.1 | 0 | 0.95 | -0.18 | 0 | 0 |
|  | *RMix_Y_* | 0 | 0.35 | 0 | 0 | 0 | 0 | 0.1 | 0.06 | 0 | 0 |
|  | *RMix_M_* | 0 | 0.17 | 0 | 0 | 0 | 0 | 0.02 | 0.1 | 0 | 0 |
|  | *RMix_S_* | 0 | 0.24 | 0 | 0 | 0 | 0 | 0.04 | 0.09 | 0 | 0 |
|  | *Sus^(IC^_Y_* | -1.99 | 0 | 0 | 0 | 0 | 0 | 0 | 0 | -1.87 | 0 |
|  | *Sus^(IC^_M_* | -1.99 | 0 | 0 | 0 | 0 | 0 | 0 | 0 | -1.89 | 0 |
|  | *Sus^(IC^_S_* | -1.99 | 0 | 0 | 0 | 0 | 0 | 0 | 0 | -1.9 | 0 |
|  | *E^(IC^_Y_* | 0 | -0.07 | 0 | -0.01 | -0.43 | 0 | 1.26 | 0 | 0 | 0.98 |
|  | *E^(IC^_M_* | 0 | -0.07 | 0 | -0.01 | -0.43 | 0 | 1.26 | 0 | -0.01 | 0.98 |
|  | *E^(IC^_S_* | 0 | -0.07 | 0 | -0.01 | -0.43 | 0 | 1.26 | 0 | -0.01 | 0.98 |
|  | *Sym^(IC^_Y_* | 0.04 | 0.85 | -0.77 | -0.01 | -0.2 | 0 | 0.98 | 0 | 0.04 | 0.91 |
|  | *Sym^(IC^_M_* | 0.04 | 0.88 | -0.92 | -0.01 | -0.28 | 0 | 1.06 | 0 | 0.03 | 0.92 |
|  | *Sym^(IC^_S_* | 0.04 | 0.89 | -0.94 | -0.01 | -0.32 | 0 | 1.1 | 0 | 0.03 | 0.93 |
|  | *H^(IC^_Y_* | 0.02 | 0.61 | 0.15 | 0 | -0.02 | 0 | 0.59 | 0 | -0.07 | 0.46 |
|  | *H^(IC^_M_* | 0.04 | 0.69 | 0.05 | 0 | -0.05 | 0 | 0.73 | 0 | -0.18 | 0.48 |
|  | *H^(IC^_S_* | 0.05 | 0.72 | 0.03 | -0.01 | -0.07 | 0 | 0.79 | 0 | -0.24 | 0.45 |
|  | *D^(IC^_Y_* | 0 | 0 | 0 | 0 | 0 | 0 | 0 | 0 | 0.23 | 0 |
|  | *D^(IC^_M_* | 0 | 0.01 | 0 | 0 | 0 | 0 | 0 | 0 | 0.26 | 0.02 |
|  | *D^(IC^_S_* | 0 | 0.02 | 0 | 0 | 0 | 0 | 0 | 0 | 0.27 | 0.04 |
| *Total effect* | | 12.2 | 18.1 | 5.9 | 3.3 | 12.8 | 2.0 | 25.8 | 5.4 | 13.9 | 7.7 |
| *Total effect immunocompetent Pop* | | 6.0 | 13.2 | 3.0 | 3.3 | 10.6 | 2.0 | 16.8 | 5.4 | 6.9 | 0.5 |
| *Total effect SIC Pop* | | 6.2 | 4.9 | 2.9 | 0.1 | 2.2 | 0.0 | 9.0 | 0.0 | 7.0 | 7.2 |
