# Supplementary material for: A dynamic model of COVID-19 infection quantifies the impact of preventive interventions on the infection of severely immunocompromised subjects in the United Kingdom
Source: PLoS One. 2026 Feb 23;21(2):e0341331. doi: 10.1371/journal.pone.0341331 (PMC12928435; doi:10.1371/journal.pone.0341331)
Supplement: S2 File — (ZIP) [file pone.0341331.s002.zip › Covid_ImmComprom_Integration.html]

Covid\_ImmComprom\_Integration


```
%%%integrates Covid_ODEs

clear all;clc;close all;

global Env Pop_T Doses Doses_out alpha_t tau_t delta_t omega_t vac1_day_end len;

%%%%%%%%%%%%%%%%%%%%%
%------load data   %%
%%%%%%%%%%%%%%%%%%%%%

load('dataTemp20_22.mat');%enviromental temperataure data
Env=Book1{1:end,{'Day' 'Date' 'Temp' }};

load('DoseDay.mat');%Vaccine dose 1,2,3,4. per week data to age groups
Doses=Doses;
%'day'	'Y1' 'M1' 'S1'	'Y2' 'M2' 'S2' 'Y3' 'M3' 'S3' 'Y4' 'M4' 'S4'


load('dataUKmatlab.mat');
%dataUK=dataUKmatlab{1:end,{'day' 'cumDeaths'	'hospitalCases'	'CasesDay' 'DeathsDay'}};
%time unit is days


%%%%%%%%%%%%%%%%%%%%%
% Parameter Values %%
%%%%%%%%%%%%%%%%%%%%%

LenImm=4.5; %months of immunity duration for infection and vaccine
LenImm_Omicron=0.5*LenImm;%stegger medrxiv indicate reinfiction hapepns before 60 dyas for omicron

Pop_Y=37.2*10^6; %<45yrs
Pop_M=23.92*10^6; %45-75 yrs
Pop_S=5.7*10^6; %>75 yrs
Pop_T=Pop_Y+Pop_M+Pop_S;

PopIC_Y=0.005*Pop_Y;% 0.5% pop with Long term diseases
PopIC_M=0.015*Pop_M;%1.5%
PopIC_S=0.025*Pop_S;%2.5%
PopIC=PopIC_Y+PopIC_M+PopIC_S;%needs to adds up to 100K whcih is num of severely IC in UK

nIC=0.007*Pop_T;% 0.7% accoridng to Evans Lancett
PopIC_Y=PopIC_Y*nIC/PopIC;% 0.5% pop with Long term diseases
PopIC_M=PopIC_M*nIC/PopIC;%1.5%
PopIC_S=PopIC_S*nIC/PopIC;%2.5%
PopIC=PopIC_Y+PopIC_M+PopIC_S;%adds up to nIC
Pop_Y=Pop_Y-PopIC_Y;
Pop_M=Pop_M-PopIC_M;
Pop_S=Pop_S-PopIC_S;

Pop=Pop_Y+Pop_M+Pop_S;
inIC_Y=PopIC_Y/180; %flow of IC population whcih is renew every 3 months
inIC_M=PopIC_M/180;
inIC_S=PopIC_S/180;
fIC_Y=PopIC_Y/nIC;
fIC_M=PopIC_M/nIC;
fIC_S=PopIC_S/nIC;

PopV=[];
PopV=[0*Pop_Y, 0*Pop_M, 0*Pop_S]; %vaccine rejection and efficacy is determine when setting up final date vaccination and rate vaccination

% model parameters
Qu=0.01; %reduction in encounters-mobility due to quanrentine it is assumed to be equal  to lam do fitted to data
tau=1/5; %incubation period
tau_Omicron=1/3;
omega=0.025;%0.0325; %rate of hospitalized from symtomatic multiplied by symtomatic fr6ction %Menachemi 2.1%. Journal of Public Health Management and Practice 27(3):p 246-250, May/June 2021. from datarate of hospitalization  Vitality indicates 3.5 from total or 6.69 per 100.000 infected according to gov.uk admisions in hospitals;0.000069*2/9.3 to account for symtomatic
omega_Omicron=omega*1; %hospital admission for omicron is 59% lower and risk of death 69 % lower comapred to delta
ro_Sym=1/10; %recovery of symtomatic ( lavezzo 9.3 days; median in vitality is 16 days )
ro_Asym=1/10;%recovery of asymtomatic and vaccinated symptomatic who do nort go to hospital
%ro_ImmCarr=1/5;%100; % clearnace rate for postive immune carriers equal to incubation period
ro_hos=(2/3)*(1/7);%recovery of hospitalized in group that recovers, 0.35 can have severe reinfection acodinly to hadley in MedArrch
delta=0.068; %rate of death in hsopitalized
delta_Delta=0.0450251905;
delta_Omicron=0.0250704;
%medic 2022 inidcates that hospitalization risk is 3 time slower for
%reinfection waht we extrapolate to say that immnity aginast sevryiy
%disease is 3 times longer
phi_ResInf=1/(30*LenImm); %1st order loss immunity rate in resistant by infection. they move to imunized suscptible
phi_ResMixed=1/(30*LenImm); %1st order loss immunity rate in resitnat by any cause they move to immunized susceptible (starts when giving the second dose)
phi_SusImm=1/(30*3*LenImm);%12 m for immunize people to have again sevre disease Medic_Lancet reference indicates hospotalizatiosn are very uncommon in reinfections of vaccinated or unvacinates peple
%all above is changed in the ODE function to vary with teh varaint
trav=0; %initially International travelling  highly restricted. Updated later

enc_Y=0.65;%encounters-mobility in young is 1.4 in midle is 0.7 and in senior is 0.35 so thsi enconuters add up to 1 and keep relation*/
enc_M=0.11;
enc_S=0.24;

% fraction hospitalizaed in young. Sum to 1 over the three ages: 2.9	percent over all Young in hsopital 19.6	percent over all middle in hsopital and 33	percent over all senior in hsopital*/
H_Y=0.01;%age factor for hospitalizatiosn form doherty*/
H_M=0.25;
H_S=0.74;

D_Y=0.1;% factor dead in young accoring  */a
D_M=0.35;
D_S=0.55;

f_Asy=0.4;%fection asymtomaitc within infected, it is 0.4 accoring to report

%all added parameters for immunocompromise subpop
Shi_IC=0.00605; %1 for no shield; 0.5 for 505 and 0.1 for 90%. reduction in mobilitye of immunocompromise because of shielding (multiplies mobility of age groups).Fitted value Not to touch
Prot0=0; %no protection
Prot_IC=0.5; %0.25 if 25% population are portected, 0.5 , o.75 or 1 for 50, 75 or 100 % protection
phi_IC=1/(30*0.1);% rate loss immunity i immunocpmpromised
delta_IC=(1/3)*(1/5);% death rate in immunocompromised infected people (28% died from hospitalized) (equal to standard pop)
omega_IC=16*omega; % rate of hospitalization in immunocomcpromised (76% of infected from review)
ro_IC=1/10; %rate infected reocvery
ro_hos_IC=(2/3)*(1/7);%rate recovery from hsopit_0l=para(34);%rate recovery from hsopital (equal to standard pop)
HIC_Y=0.10; %to fit barhemand percentage
HIC_M=0.35;
HIC_S=0.55;


 %%%%%%%%%%%%%%%%%%%%%
% Vacicnation paras %%
%%%%%%%%%%%%%%%%%%%%%

effi1_sev=1;%efficacy of first dose against severe disease
effi1_mild=0.7;%efficacy of first dose against mild disease
effi_booster=1;%efficacy booster against mild disease
timeeff_FD=21;
timeeff_PostFD=14;


 %%%%%%%%%%%%%%%%%%%%%
% Events and Variants paras %%
%%%%%%%%%%%%%%%%%%%%%
k=2; %seasonality
T_50=20;%22000;
h_T=1;

lamLock1=0.5; % lockdown effect on transmission
lamLock2=0.7;%secodn lockdown
lamSchEast22=0.5; %school holiday in 22 easter
lamSchSumm22=0.8;%school holiday 21
lamSchEast23=lamSchSumm22;

a_0=1.617480307715175191582950845908475159/Pop_T;
a_Delta=3.03841135158/Pop_T;
a_Omicron=5.3907283040/Pop_T;

t_ChDelta=279;%emmerging daya of delta variant .
t_ChOmicron=506; %emmerging daya of Omicron variant .
t_DomOmicron= t_ChOmicron;%conoslidate din january 1st
Emerg_period=45; %days from detection variant to domicnace (domiPercent)
domiPercent=0.95;
h_Delta=log(1/domiPercent-1)/log(t_ChDelta/(t_ChDelta+Emerg_period/2));
h_Omicron=log(1/domiPercent-1)/log(t_ChOmicron/(t_ChOmicron+Emerg_period/2));
%%%%%%%%%%%%%%%%%%%%%%%%%%%%%%%
% times of events %%%%%%%%%%%%%
%%%%%%%%%%%%%%%%%%%%%%%%%%%%%%%%


tstop = 1900; %Simulation days 425 is 30th september %days should be an integer

Lock1_ini= 96;%lockdown on nov 5th 2020-96, lockdown lifted on dec 2nd 2020-123,lockdown on 5th Jan 2020(157) until 12 april 2021 (254-) or 17th May 2021 (289) and vac_ini on 167+21
Lock1_end=123;
Lock2_ini= 157;%lockdown on nov 5th 2020-96, lockdown lifted on dec 2nd 2020-123,lockdown on 5th Jan 2020(157) until 12 april 2021 (254-) or 17th May 2021 (289) and vac_ini on 167+21
Lock2_end=289;
East22_ini=616;%school easter holiday 9 april
East22_end=616+15;
Holy22_ini=348+365; %school holiday from 15 July to 15 september
Holy22_end=410+365;%school holiday from 15 July to 15 september
East23_ini=973;%april 1st 23
East23_end=973+15; %april 15 23
t_trav1=366; %time to open for travelling on august 2nd 2021 with no qurentine required
t_trav2=594; %time to drop requirment of covid test 72 h pretravel for internationa; passnagers in UK airpotsr
t_Prot1ini= 440 ;%15 oct 2021 to 15 april 2022
t_Prot1end=  622;
t_Prot2ini= 805;%15 oct 2022
tchange1 = [0, Lock1_ini,Lock1_end, Lock2_ini, Lock2_end, t_trav1,t_trav2,East22_ini,East22_end, Holy22_ini,Holy22_end,t_ChOmicron,t_DomOmicron, East23_ini, East23_end,t_Prot1ini, t_Prot1end,t_Prot2ini, tstop];

lam=1; %transmission inhibition updated during sim
[tchange,order]=sort(tchange1);
lams=[]; %vector with transmission inhibition according to events
for i=1:length(tchange)%lockdown on nov 5th-96, lockdown lifted on dec 2nd-123,lockdown on 5th january (157)  and vac_ini on 167+21
    lams(i)=1;
    if (tchange(i)>=Lock1_ini && tchange(i)<Lock1_end)
        lams(i)=lamLock1;
    else
        if (tchange(i)>=Lock2_ini && tchange(i)<Lock2_end)
            lams(i)=lamLock2;
        else
            if (tchange(i)>=Holy22_ini && tchange(i)<Holy22_end)
                lams(i)=lamSchSumm22;
            else
                if (tchange(i)>=East23_ini && tchange(i)<East23_end)
                    lams(i)=lamSchEast23;
                else
                    lams(i)=1;
                end
            end
        end
    end
end

%%%%%%%%%%%%%%%%%%%%%%%%%%%%%%%%%%%
% Vectors with parameter values %%%
%%%%%%%%%%%%%%%%%%%%%%%%%%%%%%%%%%%

para=[];
para=[a_0,lam, Qu,tau, trav, ro_Asym, ro_Sym,ro_hos,delta,omega, phi_ResInf,phi_ResMixed,phi_SusImm,LenImm,enc_Y,enc_M,enc_S,...
    H_Y,H_M,H_S,D_Y,D_M,D_S,f_Asy];
para_IC=[Shi_IC,ro_IC,ro_hos_IC,inIC_Y,inIC_M,inIC_S,PopIC_Y,PopIC_M,PopIC_S,fIC_Y,fIC_M,fIC_S,HIC_Y,HIC_M, HIC_S, 1-Prot0];


VacR=[];
VacR = [effi1_sev, effi1_mild, effi_booster,timeeff_FD,timeeff_PostFD];

para_f=[];
para_f=[k, T_50, h_T];
para_Var=[];
para_Var=[t_ChDelta,t_ChOmicron, a_Delta,a_Omicron,h_Delta,h_Omicron,tau_Omicron,delta_Delta,delta_Omicron,omega_Omicron,LenImm_Omicron];

%%%%%%%%%%%%%%%%%%%%%%%%%%%%%%%
% Initial Values General Pop %%
%%%%%%%%%%%%%%%%%%%%%%%%%%%%%%%%
cumdeath_Atd0=57369;
hospital_Atd0=1291;
cumcases_Atd0=cumdeath_Atd0/(omega);%estimated from relation between cuminfection and cumdeaths in summer 2021
activeCases_Atd0=max(4611,1/f_Asy*hospital_Atd0/omega)*0.5;
IncCases_Atd0=activeCases_Atd0*5/16; %number of cases in incubation corresponding to infected at d0 5days/16days infection
resist_Atd0=0.6*cumcases_Atd0+0.5*0.4*cumcases_Atd0;%estimated from cumcases but probbaliy underestimates very much becaus elack of regsiattion of cases of frist wave
Newcases_0=4610.58;
Immu_Atd0=0.02*resist_Atd0;%assumption


Inc_0=IncCases_Atd0;
Sym_0=(1-f_Asy)*activeCases_Atd0;
Asym_0=(f_Asy)*activeCases_Atd0;
Dead_0=cumdeath_Atd0;
Resis_0=resist_Atd0;
Hosp_0=hospital_Atd0;
Sus_0=Pop-Hosp_0-Resis_0-Dead_0-Asym_0-Sym_0-Inc_0-Immu_Atd0;

Sus_Y_0=Pop_Y/Pop*Sus_0;%estimated from age percent in popoulation
Sus_M_0=Pop_M/Pop*Sus_0;
Sus_S_0=Pop_S/Pop*Sus_0;

Inc_Y_0=Pop_Y/Pop*Inc_0;
Inc_M_0=Pop_M/Pop*Inc_0;
Inc_S_0=Pop_S/Pop*Inc_0;

Sym_Y_0=Pop_Y/Pop*Sym_0; %estimated from percent of infective by age
Sym_M_0=Pop_M/Pop*Sym_0;
Sym_S_0=Pop_S/Pop*Sym_0;

Asym_Y_0=Pop_Y/Pop*Asym_0;
Asym_M_0=Pop_M/Pop*Asym_0;
Asym_S_0=Pop_S/Pop*Asym_0;

Hosp_Y_0=0.10*Hosp_0;%In hospital
Hosp_M_0=0.47*Hosp_0;
Hosp_S_0=0.43*Hosp_0;


ResInf_Y_0=Pop_Y/Pop*Resis_0;%resitant
ResInf_M_0=Pop_M/Pop*Resis_0;
ResInf_S_0=Pop_S/Pop*Resis_0;

Dead_Y_0=0.05*Dead_0;%dead
Dead_M_0=0.45*Dead_0;
Dead_S_0=0.50*Dead_0;
%initial values for immune pop
SusImm_Y_0=Pop_Y/Pop*Immu_Atd0*3/5;
SusImm_M_0=Pop_M/Pop*Immu_Atd0*3/5;
SusImm_S_0=Pop_S/Pop*Immu_Atd0*3/5;

IncImm_Y_0=Pop_Y/Pop*Immu_Atd0*1/10;
IncImm_M_0=Pop_M/Pop*Immu_Atd0*1/10;
IncImm_S_0=Pop_S/Pop*Immu_Atd0*1/10;

SymImm_Y_0=Pop_Y/Pop*Immu_Atd0*1/10;
SymImm_M_0=Pop_M/Pop*Immu_Atd0*1/10;
SymImm_S_0=Pop_S/Pop*Immu_Atd0*1/10;

AsymImm_Y_0=Pop_Y/Pop*Immu_Atd0*1/10;
AsymImm_M_0=Pop_M/Pop*Immu_Atd0*1/10;
AsymImm_S_0=Pop_S/Pop*Immu_Atd0*1/10;

ResMixed_Y_0=Pop_Y/Pop*Immu_Atd0*1/10;
ResMixed_M_0=Pop_M/Pop*Immu_Atd0*1/10;
ResMixed_S_0=Pop_S/Pop*Immu_Atd0*1/10;


%%%%%%%%%%%%%%%%%%%%%%%%%%%
% Initial Values IC Pop %%
%%%%%%%%%%%%%%%%%%%%%%%%%%%

IncIC_0=Inc_0/Pop*PopIC*Shi_IC;
SymIC_0=Sym_0/Pop*PopIC*Shi_IC;
ResisIC_0=0;
HospIC_0=Hosp_0/Pop*PopIC;
DeadIC_0=Dead_0/Pop*PopIC;
SusIC_0=PopIC-HospIC_0-ResisIC_0-DeadIC_0-SymIC_0-IncIC_0;

SusIC_Y_0=fIC_Y*SusIC_0;
SusIC_M_0=fIC_M*SusIC_0;
SusIC_S_0=fIC_S*SusIC_0;

IncIC_Y_0=PopIC_Y/PopIC*IncIC_0;
IncIC_M_0=PopIC_M/PopIC*IncIC_0;
IncIC_S_0=PopIC_S/PopIC*IncIC_0;

SymIC_Y_0=fIC_Y*SymIC_0;
SymIC_M_0=fIC_M*SymIC_0;
SymIC_S_0=fIC_S*SymIC_0;

HospIC_Y_0=fIC_Y*HospIC_0;
HospIC_M_0=fIC_M*HospIC_0;
HospIC_S_0=fIC_S*HospIC_0;

ResIC_Y_0=0;
ResIC_M_0=0;
ResIC_S_0=0;

DeadIC_Y_0=fIC_Y*DeadIC_0;
DeadIC_M_0=fIC_M*DeadIC_0;
DeadIC_S_0=fIC_S*DeadIC_0;

Newcases_Y_0=enc_Y*Newcases_0;
Newcases_M_0=enc_M*Newcases_0;
Newcases_S_0=enc_S*Newcases_0;

NewcasesIC_Y_0=Newcases_Y_0/Pop*PopIC;
NewcasesIC_M_0=Newcases_M_0/Pop*PopIC;
NewcasesIC_S_0=Newcases_S_0/Pop*PopIC;

Admin_Y_0=Hosp_Y_0/7;
Admin_M_0=Hosp_M_0/7;
Admin_S_0=Hosp_S_0/7;

AdminIC_Y_0=Admin_Y_0/Pop*PopIC;
AdminIC_M_0=Admin_M_0/Pop*PopIC;
AdminIC_S_0=Admin_S_0/Pop*PopIC;
FD_Y_0=0;

%%%%%%%%%%%%%%%%%%%%%
% Vector Initial Values %%
%%%%%%%%%%%%%%%%%%%%%
 y0=[];
 y0 = [Sus_Y_0, Sus_M_0,Sus_S_0, Inc_Y_0, Inc_M_0,Inc_S_0, ...%1-6
     Sym_Y_0, Sym_M_0, Sym_S_0, Asym_Y_0, Asym_M_0, Asym_S_0, ... %7-12
        Hosp_Y_0, Hosp_M_0, Hosp_S_0, ResInf_Y_0, ResInf_M_0, ResInf_S_0, ...13-18
        Dead_Y_0, Dead_M_0, Dead_S_0, SusImm_Y_0, SusImm_M_0, SusImm_S_0, ...%19-24
        IncImm_Y_0, IncImm_M_0, IncImm_S_0, SymImm_Y_0, SymImm_M_0, SymImm_S_0, ...%25-30
        AsymImm_Y_0, AsymImm_M_0, AsymImm_S_0, ResMixed_Y_0, ResMixed_M_0, ResMixed_S_0,...%31-36
        SusIC_Y_0, SusIC_M_0, SusIC_S_0, IncIC_Y_0, IncIC_M_0, IncIC_S_0,...%37-42
        SymIC_Y_0, SymIC_M_0, SymIC_S_0, HospIC_Y_0, HospIC_M_0, HospIC_S_0,...%43-48
        ResIC_Y_0, ResIC_M_0, ResIC_S_0, DeadIC_Y_0, DeadIC_M_0, DeadIC_S_0,...%49-54
        Newcases_Y_0, Newcases_M_0, Newcases_S_0, NewcasesIC_Y_0, NewcasesIC_M_0, NewcasesIC_S_0,...%55-60
        Admin_Y_0, Admin_M_0, Admin_S_0, AdminIC_Y_0, AdminIC_M_0, AdminIC_S_0];  %66

 Plop=sum(y0(1:54))-Pop_T;
 P1=sum(y0(1:21));
 P2=sum(y0(22:36));
 P3=sum(y0(37:54));
 aa=P1+P2+P3;


%%%%%%%%%%%%%%%%%%%%%%%%%%%%%
%%%    Simulation %%%%%%%%%%
%%%%%%%%%%%%%%%%%%%%%%%%%%%%%%
tstep=1;
tPoints=[];
Points=[];
vac_rate_day=[];

jevent=1;
Currtime=tchange(jevent);
check_conservationPop1=[];
while Currtime<tstop
    while abs(( tchange(jevent+1))-Currtime) <10^-8 && Currtime<tstop &&jevent+1 <= length(tchange)
        jevent=jevent+1;
    end
    if ( tchange(jevent+1))-Currtime <1
        aux =(tchange(jevent+1)-Currtime)/2;
        tsp = Currtime: aux :( tchange(jevent+1));
    else
        tsp = Currtime: tstep :( tchange(jevent+1));
    end
    para(2)=lams(jevent);


    if  tchange(jevent)>=t_trav1
           aux=0.0004*0.5;
           para(5)=aux*250*10^6/356/Pop;  %travelling effect on transmission
    end
    if  tchange(jevent)>=t_trav2
           aux=0.002*0.5; %proportion of trvaeller infected*proportion of travels
           para(5)=aux*250*10^6/356/Pop;  %travellers opening after all population , aux is proportion of treveleers infecte
    end

     if  tchange(jevent)==t_Prot1ini  para_IC(19)=1-Prot_IC; end
     if  tchange(jevent)==t_Prot1end  para_IC(19)=1-Prot0; end
     if  tchange(jevent)==t_Prot2ini  para_IC(19)=1-Prot_IC; end


    t=[];
    y=[];
    % numerical integration
    options = odeset('RelTol', 1e-4, 'NonNegative', 1:66);
    [t,y] = ode45(@(t,y)Covid_ImmComprom_ODEs(t,y,para,para_f,para_Var,para_IC, VacR),tsp, y0, options);
    check_conservationPop1=sum(y(:,1:54),2);

    y0 = y(end, :);

    y0(y0<0)=0;
    y0=round(y0,0);

    %collecting RESULTS
    tPoints = [tPoints(1:end-1,:);t]; %ads new t vectot to tPoints
    Points=[Points(1:end-1,:); y];
    Currtime=tchange(jevent+1);
end


%%%%%%%%%%%%%%%%%%%%%%%%%%%%%
%%%    Plotting %%%%%%%%%%
%%%%%%%%%%%%%%%%%%%%%%%%%%%%%%
%Organizing results to plot

TotalsNaive=[];
for i=1:3:21
     aux=sum(Points(:,(i:i+2)),2);
     TotalsNaive=[TotalsNaive aux];
end
TotalsImm=[];
for i=22:3:36
     aux=sum(Points(:,(i:i+2)),2);
     TotalsImm=[TotalsImm aux];
end

TotalsIC=[];
for i=37:3:54
     aux=sum(Points(:,(i:i+2)),2);
     TotalsIC=[TotalsIC aux];
end

Totals=[TotalsNaive(:, (1:3))+TotalsIC(:, (1:3))+TotalsImm(:, (1:3)),TotalsNaive(:, 4)+TotalsImm(:, 4),...
            TotalsNaive(:,5)+TotalsIC(:,4),TotalsNaive(:, 6)+TotalsIC(:, 5)+TotalsImm(:, 5),TotalsNaive(:,7)+TotalsIC(:,5)];
% sus, inc, sym, asym, hosp, res, death

NewC=sum(Points(:,55:60),2);
auxNewCases=NewC(2:end,1)-NewC(1:end-1,1);

DeathsD=sum(Points(:,19:21),2)+sum(Points(:,52:54),2);
auxDeaths=DeathsD(2:end,1)-DeathsD(1:end-1,1);

Totals=[Totals [0; auxNewCases] [0;auxDeaths]];%"Sus","Inc", "Sym","Asym", "Hosp", "resistant","Dead" "newcases" "dayly deaths"

nameNaive=[];
nameNaive=["Sus","Inc", "Sym","Asym", "Hosp", "Res_Inf","Dead","Admin"];
nameImm=[];
nameImm=["SusImm","IncImm", "SymImm","AsymImm","ResMixed"];
nameIC=[];
nameIC=["SusIC","IncIC", "SymIC", "HospIC", "Res_IC","DeadIC","AdminIC"];

 check_conservationPop=sum(Points(:,1:54),2);


%%%%% PLOTTING %%%
figure("Name","Naive Pop");
%title("Naive Pop");
for splot=1:7
        subplot(2,4,splot);
       for age=1:3
            plot(tPoints,log10(Points(:,(splot-1)*3+age)+1));
            hold on
       end
        if splot==1 legend( "Y", "M","S"); end
        xlabel("days");
        ylabel("subjects");
        title(nameNaive(splot));
        hold off

end
     subplot(2,4,8);%adding Hopsital adminsiosn
     for age=1:3
            plot(tPoints,log10(Points(:,60+age)+1));
            hold on
       end
        if splot==1 legend( "Y", "M","S"); end
        xlabel("days");
        ylabel("subjects");
        title(nameNaive(8));
        hold off


figure("Name","ImmComprom Pop");
for splot=1:6
        subplot(2,4,splot);
                   for age=1:3
                        plot(tPoints,log10(Points(:,36+(splot-1)*3+age)+1));
                        hold on
                    end
                    if splot==1 legend( "Y", "M","S"); end
                    xlabel("days");
                    ylabel("subjects");
                    title(nameIC(splot));
                    hold off

end
      subplot(2,4,7);%adding Hopsital adminsiosn
     for age=1:3
            plot(tPoints,log10(Points(:,63+age)+1));
            hold on
       end
        if splot==1 legend( "Y", "M","S"); end
        xlabel("days");
        ylabel("subjects");
        title(nameIC(7));
        hold off

figure('Name', "immnunized pop");
for splot=1:5
        subplot(2,3,splot);
                   for age=1:3
                        plot(tPoints,log10(Points(:,21+(splot-1)*3+age)+1));
                        hold on
                    end
                    if splot==1 legend( "Y", "M","S"); end
                    xlabel("days");
                    ylabel("subjects");
                    title(nameImm(splot));
                    hold off

end


tplot=850;
figure ('Name', 'Pred vs Obs')
subplot(1,3,1);
datePoints=datetime(2020,8,1)+caldays(tPoints);
semilogy (datePoints,(Totals(:,5)+1),'-r',LineWidth=1);
hold on
datePoints=datetime(2020,8,1)+caldays(dataUK(:,1));
semilogy (datePoints,(dataUK(:,3)+1),'o','MarkerSize',3,'MarkerEdgeColor','blue');
hold off
title('Hospital');
xlabel('days');
xlim([datetime(2020,8,1),datetime(2022,12,1)]);
tickies=datetime(2020,8,1)+calmonths([0:2:60]);
xticks([tickies]);
ylabel('num');

subplot(1,3,2);
datePoints=datetime(2020,8,1)+caldays(tPoints);
semilogy (datePoints,(Totals(:,9)+1),'-r',LineWidth=1);
hold on
datePoints=datetime(2020,8,1)+caldays(dataUK(:,1));
semilogy(datePoints,(dataUK(:,5)+1),'o','MarkerSize',3,'MarkerEdgeColor','blue');
hold off
title('Daily deaths');
xlabel('days');
xlim([datetime(2020,8,1),datetime(2022,12,1)]);
tickies=datetime(2020,8,1)+calmonths([0:2:60]);
xticks([tickies]);
ylabel('num');

subplot(1,3,3);
datePoints=datetime(2020,8,1)+caldays(tPoints);
semilogy (datePoints,Totals(:,8),'-r',LineWidth=1);
hold on
datePoints=datetime(2020,8,1)+caldays(dataUK(:,1));
semilogy(datePoints,dataUK(:,4),'o','MarkerSize',3,'MarkerEdgeColor','blue');
hold off
title ('New Cases');
xlabel('days');
xlim([datetime(2020,8,1),datetime(2022,12,1)]);
tickies=datetime(2020,8,1)+calmonths([0:2:60]);
xticks([tickies]);
ylabel('num');
legend('Pred', 'Obs');
```

   

Published with MATLAB® R2024b
