# Supplementary material for: A dynamic model of COVID-19 infection quantifies the impact of preventive interventions on the infection of severely immunocompromised subjects in the United Kingdom
Source: PLoS One. 2026 Feb 23;21(2):e0341331. doi: 10.1371/journal.pone.0341331 (PMC12928435; doi:10.1371/journal.pone.0341331)
Supplement: S2 File — (ZIP) [file pone.0341331.s002.zip › Covid_ImmComprom_ODEs.html]

Covid\_ImmComprom\_ODEs


```
function dydt = Covid_incub_Doses_Comprom_ODEs(t,y,para,para_f,para_Var, para_IC, VacR)
global Env alpha_t tau_t omega_t delta_t Doses Doses_out len;
%from vector to local params

a_0=para(1);%infection rate
lam=para(2);%lockdown effect
Qu=para(3); %reduction in encounters due to quanrentine
tau_0=para(4);%incubation rate
trav=para(5);%travelling
ro_Asym=para(6); % recovery of asymtomatic
ro_Sym=para(7);%recovery of symtomatic
ro_hos=para(8);%recovery of hospitalized
delta_0=para(9) ; %rate of death
omega_0=para(10); %rate of hospitalization
phi_ResInf=para(11); %loss immunity rate in resistant by infection population
phi_ResMixed=para(12); %loss immunity rate in vaccinated and resistant by infection population
phi_SusImm=para(13);%loss immunity rate in immunized population resistant to severe disease only
LenImm=para(14);

enc_Y=para(15);%encounters-mobility in young
enc_M=para(16);
enc_S=para(17);

H_Y=para(18);% hospitalization in young.
H_M=para(19);
H_S=para(20);

D_Y=para(21);% death in young.
D_M=para(22);
D_S=para(23);

f_Asy=para(24);% asymtomatic within infected,

%immunocompromised pop parameters
Shi_IC=para_IC(1); %reduction in transmission of immunocompromise because of shielding
ro_IC=para_IC(2); %recovery from infection
ro_hos_IC=para_IC(3);%rate recovery from hsopital
inIC_Y=para_IC(4);
inIC_M=para_IC(5);
inIC_S=para_IC(6);
PopICss_Y=para_IC(7);
PopICss_M=para_IC(8);
PopICss_S=para_IC(9);
fIC_Y=para_IC(10);
fIC_M=para_IC(11);
fIC_S=para_IC(12);
HIC_Y=para_IC(13);
HIC_M=para_IC(14);
HIC_S=para_IC(15);
Prot_IC_epsilon=para_IC(16);

%env params
k=para_f(1);
T_50=para_f(2);
h_T=para_f(3);

%Variant paras
t_ChDelta=para_Var(1);
t_ChOmicron=para_Var(2);
a_Delta=para_Var(3);
a_Omicron=para_Var(4);
h_Delta=para_Var(5);
h_Omicron=para_Var(6);
tau_Omicron=para_Var(7);
delta_Delta=para_Var(8) ; %rate of death
delta_Omicron=para_Var(9) ; %rate of death
omega_Omicron=para_Var(10);
LenImm_Omicron=para_Var(11);


%state Vars
    Sus_Y=y(1);
    Sus_M=y(2);
    Sus_S=y(3);
    Inc_Y=y(4);
    Inc_M=y(5);
    Inc_S=y(6);
    Sym_Y=y(7);
    Sym_M=y(8);
    Sym_S=y(9);
    Asym_Y=y(10);
    Asym_M=y(11);
    Asym_S=y(12);
    Hosp_Y=y(13);
    Hosp_M=y(14);
    Hosp_S=y(15);
    ResInf_Y=y(16);
    ResInf_M=y(17);
    ResInf_S=y(18);
    Dead_Y=y(19);
    Dead_M=y(20);
    Dead_S=y(21);
    SusImm_Y=y(22);
    SusImm_M=y(23);
    SusImm_S=y(24);
    IncImm_Y=y(25);
    IncImm_M=y(26);
    IncImm_S=y(27);
    SymImm_Y=y(28);
    SymImm_M=y(29);
    SymImm_S=y(30);
    AsymImm_Y=y(31);
    AsymImm_M=y(32);
    AsymImm_S=y(33);
    ResMixed_Y=y(34);
    ResMixed_M=y(35);
    ResMixed_S=y(36);
    SusIC_Y=y(37);
    SusIC_M=y(38);
    SusIC_S=y(39);
    IncIC_Y=y(40);
    IncIC_M=y(41);
    IncIC_S=y(42);
    SymIC_Y=y(43);
    SymIC_M=y(44);
    SymIC_S=y(45);
    HospIC_Y=y(46);
    HospIC_M=y(47);
    HospIC_S=y(48);
    ResIC_Y=y(49);
    ResIC_M=y(50);
    ResIC_S=y(51);
    DeadIC_Y=y(52);
    DeadIC_M=y(53);
    DeadIC_S=y(54);
    Newcases_Y=y(55);
    Newcases_M=y(56);
    Newcases_S=y(57);
    NewcasesIC_Y=y(58);
    NewcasesIC_M=y(59);
    NewcasesIC_S=y(60);
    Admin_Y=y(61);
    Admin_M=y(62);
    Admin_S=y(63);
    AdminIC_Y=y(64);
    AdminIC_M=y(65);
    AdminIC_S=y(66);


%Vaccine para
effi1_sev=VacR(1);%efficacy agind sevre disease (here  what we do is keeping (1-sev)* pop as not immunized susceptible
effi1_mild=VacR(2); % with first dose effi_sev*Pop is immunized vs sevre but only 0,7*Pop is ummninize vs mild. So effi1* effi_sev*Pop was to resistant and (1-effi1)* effi_sev*Pop to immune susceptible
effi_booster=VacR(3);%efficacy against  mild disease in immunized people
timeeff_FD=VacR(4);
timeeff_PostFD=VacR(5);

%1st dose vaccine
TimeEff1=Doses(:,1)+timeeff_FD; %time for effectiev immunity after dose 1
TimeEff2=Doses(:,1)+timeeff_PostFD; %tiem for effective immunity after other doses

Vac1Sus_Y=0; Vac1Asym_Y=0; Vac1ResInf_Y=0; Vac1Sus_M=0; Vac1Asym_M=0; Vac1ResInf_M=0; Vac1Sus_S=0; Vac1Asym_S=0; Vac1ResInf_S=0;
BoosterSusImm_Y=0; BoosterAsymImm_Y=0; BoosterSusImm_M=0; BoosterAsymImm_M=0;  BoosterSusImm_S=0; BoosterAsymImm_S=0;

YesVac="Y"; %if  vaccination is happening
Fdose_Y=interp1(TimeEff1,Doses(:,3),t); %moving effiSev=85% of vaccinated population to immunized compartmnet
Aux_Y=Sus_Y+Asym_Y+ResInf_Y;
if Fdose_Y>0 && Aux_Y>0 && YesVac=="Y"
    Vac1Sus_Y=effi1_sev*Fdose_Y*Sus_Y/Aux_Y; % cosntnt absolute rate immunixation for first dose. Moves subjects from sus to Immne Vaccinates and from sus to sus1D
     Vac1Asym_Y=effi1_sev*Fdose_Y*Asym_Y/Aux_Y;
     Vac1ResInf_Y=effi1_sev*Fdose_Y*ResInf_Y/Aux_Y;
end

Fdose_M=interp1(TimeEff1,Doses(:,4),t); %moving effiSev=85% of vaccinated population to immunized compartmnet
Aux_M=Sus_M+Asym_M+ResInf_M;
if Fdose_M>0 && Aux_M>0 && YesVac=="Y"
    Vac1Sus_M=effi1_sev*Fdose_M*Sus_M/Aux_M; % cosntnt absolute rate immunixation for first dose. Moves subjects from sus to Immne Vaccinates and from sus to sus1D
     Vac1Asym_M=effi1_sev*Fdose_M*Asym_M/Aux_M;
     Vac1ResInf_M=effi1_sev*Fdose_M*ResInf_M/Aux_M;
end

Fdose_S=interp1(TimeEff1,Doses(:,5),t); %moving effiSev=85% of vaccinated population to immunized compartmnet
Aux_S=Sus_S+Asym_S+ResInf_S;
if Fdose_S>0 && Aux_S>0 && YesVac=="Y"
    Vac1Sus_S=effi1_sev*Fdose_S*Sus_S/Aux_S; % cosntnt absolute rate immunixation for first dose. Moves subjects from sus to Immne Vaccinates and from sus to sus1D
     Vac1Asym_S=effi1_sev*Fdose_S*Asym_S/Aux_S;
     Vac1ResInf_S=effi1_sev*Fdose_S*ResInf_S/Aux_S;
end


%vaccination of immunized population (booster)
Booster_Y=interp1(TimeEff2,Doses(:,6),t)+interp1(TimeEff2,Doses(:,6+3),t)+interp1(TimeEff2,Doses(:,6+6),t);
Aux_Y=SusImm_Y+AsymImm_Y+ResMixed_Y;    %we osnsider only effiSev&pop respons to firs and secodn dose
if Booster_Y>0 && Aux_Y>0 && YesVac=="Y"
    BoosterSusImm_Y=effi_booster*Booster_Y*SusImm_Y/Aux_Y; % cosntnt absolute rate immunixation for first dose. Moves subjects from sus to Immne Vaccinates and from sus to sus1D
    BoosterAsymImm_Y=effi_booster*Booster_Y*AsymImm_Y/Aux_Y;
    %ResMixed_Y IS already immunized
end

Booster_M=interp1(TimeEff2,Doses(:,7),t)+interp1(TimeEff2,Doses(:,7+3),t)+interp1(TimeEff2,Doses(:,7+6),t);
Aux_M=SusImm_M+AsymImm_M+ResMixed_M;    %we osnsider only effiSev&pop respons to first and second dose
if Booster_M>0 && Aux_M>0 && YesVac=="Y"
    BoosterSusImm_M=effi_booster*Booster_M*SusImm_M/Aux_M; % cosntnt absolute rate immunixation for first dose. Moves subjects from sus to Immne Vaccinates and from sus to sus1D
    BoosterAsymImm_M=effi_booster*Booster_M*AsymImm_M/Aux_M;
    %ResMixed_M IS already immunized
end

Booster_S=interp1(TimeEff2,Doses(:,8),t)+interp1(TimeEff2,Doses(:,8+3),t)+interp1(TimeEff2,Doses(:,8+6),t);
Aux_S=SusImm_S+AsymImm_S+ResMixed_S;    %we osnsider only effiSev&pop respons to firs and secodn dose
if Booster_S>0 && Aux_S>0 && YesVac=="Y"
    BoosterSusImm_S=effi_booster*Booster_S*SusImm_S/Aux_S; % cosntnt absolute rate immunixation for first dose. Moves subjects from sus to Immne Vaccinates and from sus to sus1D
    BoosterAsymImm_S=effi_booster*Booster_S*AsymImm_S/Aux_S;
    %ResMixed_S IS already immunized
end

Doses_out=[Doses_out; Vac1Sus_Y, Vac1Asym_Y, Vac1ResInf_Y, Vac1Sus_M, Vac1Asym_M, Vac1ResInf_M, Vac1Sus_S, Vac1Asym_S, Vac1ResInf_S...
BoosterSusImm_Y, BoosterAsymImm_Y, BoosterSusImm_M, BoosterAsymImm_M,  BoosterSusImm_S, BoosterAsymImm_S];


%interpolate temperature
Ef1=interp1(Env(:,1),Env(:,3),t);% 3 is temp and 4 is UV for Noseasonaluty in 2021 5 is temp and 6 is UV

%adjusting Transmission rate according to variant
    alpha=a_0;
    t_ChOmicron_0=t_ChOmicron; %time of Omicron emregence
    alpha=a_0+t^h_Delta/(t^h_Delta+t_ChDelta^h_Delta)*(a_Delta-a_0)+t^h_Omicron/(t^h_Omicron+t_ChOmicron^h_Omicron)*(a_Omicron-a_Delta);
    alpha_t= [alpha_t; t, alpha ];

%adjusting death rate according to variant
    t_aux_D=t_ChDelta+20;
    t_aux_O=t_ChOmicron+20;
    delta=delta_0+t^h_Delta/(t^h_Delta+t_aux_D^h_Delta)*(delta_Delta-delta_0)+t^h_Omicron/(t^h_Omicron+t_aux_O^h_Omicron)*(delta_Omicron-delta_Delta);
    delta_IC=1.02*delta; %adjusting death rate according to variant in IC patients
    delta_t= [delta_t; t, delta ];

   if (t>t_ChOmicron_0+60) && (t<t_ChOmicron_0+65)
         phi_ResInf=5*1/(30*LenImm_Omicron);
          phi_ResMixed=5*1/(30*LenImm_Omicron);
           phi_SusImm=1/(30*3*LenImm_Omicron);
   else
         if (t>t_ChOmicron_0+LenImm*30/2)
                phi_ResInf=1/(30*LenImm); %1st order loss immunity rate in resistant by infection. they move to imunized suscptible
                phi_ResMixed=1/(30*LenImm); %1st order loss immunity rate in resitnat by any cause they move to immunized susceptible (starts when giving the second dose)
                phi_SusImm=1/(30*3*LenImm);%12 m for immunize people to have again sevre disease Medic_Lancet reference indicates hospotalizatiosn are very uncommon in reinfections of vaccinated or unvacinates peple
         else
            phi_ResInf=1/(30*LenImm); %1st order loss immunity rate in resistant by infection. they move to imunized suscptible
              phi_ResMixed=1/(30*LenImm); %1st order loss immunity rate in resitnat by any cause they move to immunized susceptible (starts when giving the second dose)
               phi_SusImm=1/(30*3*LenImm);%12 m for immunize people to have again sevre disease Medic_Lancet reference indicates hospotalizatiosn are very uncommon in reinfections of vaccinated or unvacinates peple
         end
   end

%adjusting incubation time according to variant
    tau=tau_0+(tau_Omicron-tau_0)*t^h_Omicron/(t^h_Omicron+t_ChOmicron^h_Omicron);
    tau_t= [tau_t; t, tau ];
%adjusting hospitalization rate according to variant
    t_ChOmegaOmicron=t_ChOmicron+15;
    omega=omega_0+(omega_Omicron-omega_0)*t^h_Omicron/(t^h_Omicron+t_ChOmegaOmicron^h_Omicron);
    omega_IC=omega*16;
    omega_t= [omega_t; t, omega ];


%ODEs
     %Immunocompromised compartments
    dSusIC_Y=-alpha*lam*f_env_T50(Ef1,k,T_50,h_T)*Qu*(enc_Y*Sym_Y+enc_M*Sym_M+enc_S*Sym_S)*Shi_IC*Prot_IC_epsilon*SusIC_Y-alpha*lam*f_env_T50(Ef1,k,T_50,h_T)*(enc_Y*Asym_Y+enc_M*Asym_M+enc_S*Asym_S)*Shi_IC*Prot_IC_epsilon*SusIC_Y ...
            -alpha*lam*f_env_T50(Ef1,k,T_50,h_T)*Qu*(enc_Y*SymImm_Y+enc_M*SymImm_M+enc_S*SymImm_S)*Shi_IC*Prot_IC_epsilon*SusIC_Y-alpha*lam*f_env_T50(Ef1,k,T_50,h_T)*(enc_Y*AsymImm_Y+enc_M*AsymImm_M+enc_S*AsymImm_S)*Shi_IC*Prot_IC_epsilon*SusIC_Y ...
             -alpha*lam*f_env_T50(Ef1,k,T_50,h_T)*Qu*Shi_IC*(enc_Y*SymIC_Y+enc_M*SymIC_M+enc_S*SymIC_S)*Shi_IC*Prot_IC_epsilon*SusIC_Y...
                +ro_IC*SymIC_Y+ro_hos_IC*HospIC_Y+inIC_Y-inIC_Y/PopICss_Y*SusIC_Y;
    dSusIC_M=-alpha*lam*f_env_T50(Ef1,k,T_50,h_T)*Qu*(enc_Y*Sym_Y+enc_M*Sym_M+enc_S*Sym_S)*Shi_IC*Prot_IC_epsilon*SusIC_M-alpha*lam*f_env_T50(Ef1,k,T_50,h_T)*(enc_Y*Asym_Y+enc_M*Asym_M+enc_S*Asym_S)*Shi_IC*Prot_IC_epsilon*SusIC_M ...
             -alpha*lam*f_env_T50(Ef1,k,T_50,h_T)*Qu*(enc_Y*SymImm_Y+enc_M*SymImm_M+enc_S*SymImm_S)*Shi_IC*Prot_IC_epsilon*SusIC_M-alpha*lam*f_env_T50(Ef1,k,T_50,h_T)*(enc_Y*AsymImm_Y+enc_M*AsymImm_M+enc_S*AsymImm_S)*Shi_IC*Prot_IC_epsilon*SusIC_M ...
              -alpha*lam*f_env_T50(Ef1,k,T_50,h_T)*Qu*Shi_IC*(enc_Y*SymIC_Y+enc_M*SymIC_M+enc_S*SymIC_S)*Shi_IC*Prot_IC_epsilon*SusIC_M...
             +ro_IC*SymIC_M+ro_hos_IC*HospIC_M+inIC_M-inIC_M/PopICss_M*SusIC_M;
    dSusIC_S=-alpha*lam*f_env_T50(Ef1,k,T_50,h_T)*Qu*(enc_Y*Sym_Y+enc_M*Sym_M+enc_S*Sym_S)*Shi_IC*Prot_IC_epsilon*SusIC_S-alpha*lam*f_env_T50(Ef1,k,T_50,h_T)*(enc_Y*Asym_Y+enc_M*Asym_M+enc_S*Asym_S)*Shi_IC*Prot_IC_epsilon*SusIC_S ...
            -alpha*lam*f_env_T50(Ef1,k,T_50,h_T)*Qu*(enc_Y*SymImm_Y+enc_M*SymImm_M+enc_S*SymImm_S)*Shi_IC*Prot_IC_epsilon*SusIC_S-alpha*lam*f_env_T50(Ef1,k,T_50,h_T)*(enc_Y*AsymImm_Y+enc_M*AsymImm_M+enc_S*AsymImm_S)*Shi_IC*Prot_IC_epsilon*SusIC_S ...
            -alpha*lam*f_env_T50(Ef1,k,T_50,h_T)*Qu*Shi_IC*(enc_Y*SymIC_Y+enc_M*SymIC_M+enc_S*SymIC_S)*Shi_IC*Prot_IC_epsilon*SusIC_S...
            +ro_IC*SymIC_S+ro_hos_IC*HospIC_S+inIC_S-inIC_S/PopICss_S*SusIC_S;


    dIncIC_Y=+alpha*lam*f_env_T50(Ef1,k,T_50,h_T)*Qu*(enc_Y*Sym_Y+enc_M*Sym_M+enc_S*Sym_S)*Shi_IC*Prot_IC_epsilon*SusIC_Y+alpha*lam*f_env_T50(Ef1,k,T_50,h_T)*(enc_Y*Asym_Y+enc_M*Asym_M+enc_S*Asym_S)*Shi_IC*Prot_IC_epsilon*SusIC_Y ...
            +alpha*lam*f_env_T50(Ef1,k,T_50,h_T)*Qu*(enc_Y*SymImm_Y+enc_M*SymImm_M+enc_S*SymImm_S)*Shi_IC*Prot_IC_epsilon*SusIC_Y+alpha*lam*f_env_T50(Ef1,k,T_50,h_T)*(enc_Y*AsymImm_Y+enc_M*AsymImm_M+enc_S*AsymImm_S)*Shi_IC*Prot_IC_epsilon*SusIC_Y ...
             +alpha*lam*f_env_T50(Ef1,k,T_50,h_T)*Qu*Shi_IC*(enc_Y*SymIC_Y+enc_M*SymIC_M+enc_S*SymIC_S)*Shi_IC*Prot_IC_epsilon*SusIC_Y...
               -tau*IncIC_Y;
    dIncIC_M=+alpha*lam*f_env_T50(Ef1,k,T_50,h_T)*Qu*(enc_Y*Sym_Y+enc_M*Sym_M+enc_S*Sym_S)*Shi_IC*Prot_IC_epsilon*SusIC_M+alpha*lam*f_env_T50(Ef1,k,T_50,h_T)*(enc_Y*Asym_Y+enc_M*Asym_M+enc_S*Asym_S)*Shi_IC*Prot_IC_epsilon*SusIC_M ...
             +alpha*lam*f_env_T50(Ef1,k,T_50,h_T)*Qu*(enc_Y*SymImm_Y+enc_M*SymImm_M+enc_S*SymImm_S)*Shi_IC*Prot_IC_epsilon*SusIC_M+alpha*lam*f_env_T50(Ef1,k,T_50,h_T)*(enc_Y*AsymImm_Y+enc_M*AsymImm_M+enc_S*AsymImm_S)*Shi_IC*Prot_IC_epsilon*SusIC_M ...
              +alpha*lam*f_env_T50(Ef1,k,T_50,h_T)*Qu*Shi_IC*(enc_Y*SymIC_Y+enc_M*SymIC_M+enc_S*SymIC_S)*Shi_IC*Prot_IC_epsilon*SusIC_M...
                   -tau*IncIC_M;
    dIncIC_S=+alpha*lam*f_env_T50(Ef1,k,T_50,h_T)*Qu*(enc_Y*Sym_Y+enc_M*Sym_M+enc_S*Sym_S)*Shi_IC*Prot_IC_epsilon*SusIC_S+alpha*lam*f_env_T50(Ef1,k,T_50,h_T)*(enc_Y*Asym_Y+enc_M*Asym_M+enc_S*Asym_S)*Shi_IC*Prot_IC_epsilon*SusIC_S ...
            +alpha*lam*f_env_T50(Ef1,k,T_50,h_T)*Qu*(enc_Y*SymImm_Y+enc_M*SymImm_M+enc_S*SymImm_S)*Shi_IC*Prot_IC_epsilon*SusIC_S+alpha*lam*f_env_T50(Ef1,k,T_50,h_T)*(enc_Y*AsymImm_Y+enc_M*AsymImm_M+enc_S*AsymImm_S)*Shi_IC*Prot_IC_epsilon*SusIC_S ...
            +alpha*lam*f_env_T50(Ef1,k,T_50,h_T)*Qu*Shi_IC*(enc_Y*SymIC_Y+enc_M*SymIC_M+enc_S*SymIC_S)*Shi_IC*Prot_IC_epsilon*SusIC_S...
            -tau*IncIC_S;

    dSymIC_Y=tau*IncIC_Y-omega_IC*HIC_Y*SymIC_Y-ro_IC*SymIC_Y;
    dSymIC_M=tau*IncIC_M-omega_IC*HIC_M*SymIC_M-ro_IC*SymIC_M;
    dSymIC_S=tau*IncIC_S-omega_IC*HIC_S*SymIC_S-ro_IC*SymIC_S;


    dHospIC_Y=omega_IC*HIC_Y*SymIC_Y-ro_hos_IC*HospIC_Y-delta_IC*D_Y*HospIC_Y;
    dHospIC_M=omega_IC*HIC_M*SymIC_M-ro_hos_IC*HospIC_M-delta_IC*D_M*HospIC_M;
    dHospIC_S=omega_IC*HIC_S*SymIC_S-ro_hos_IC*HospIC_S-delta_IC*D_S*HospIC_S;


    dResIC_Y=0;
    dResIC_M=0;
    dResIC_S=0;

    dDeadIC_Y=delta_IC*D_Y*HospIC_Y;
    dDeadIC_M=delta_IC*D_M*HospIC_M;
    dDeadIC_S=delta_IC*D_S*HospIC_S;


    %Naive Pop

    dSus_Y=-alpha*lam*f_env_T50(Ef1,k,T_50,h_T)*Qu*(enc_Y*Sym_Y+enc_M*Sym_M+enc_S*Sym_S)*enc_Y*Sus_Y-alpha*lam*f_env_T50(Ef1,k,T_50,h_T)*(enc_Y*Asym_Y+enc_M*Asym_M+enc_S*Asym_S)*enc_Y*Sus_Y ...
            -alpha*lam*f_env_T50(Ef1,k,T_50,h_T)*Qu*(enc_Y*SymImm_Y+enc_M*SymImm_M+enc_S*SymImm_S)*enc_Y*Sus_Y-alpha*lam*f_env_T50(Ef1,k,T_50,h_T)*(enc_Y*AsymImm_Y+enc_M*AsymImm_M+enc_S*AsymImm_S)*enc_Y*Sus_Y ...
              -alpha*lam*f_env_T50(Ef1,k,T_50,h_T)*Qu*Shi_IC*(enc_Y*SymIC_Y+enc_M*SymIC_M+enc_S*SymIC_S)*enc_Y*Sus_Y...
              -trav*Sus_Y-Vac1Sus_Y+phi_SusImm*SusImm_Y...
              -inIC_Y+inIC_Y/PopICss_Y*SusIC_Y;
    dSus_M=-alpha*lam*f_env_T50(Ef1,k,T_50,h_T)*Qu*(enc_Y*Sym_Y+enc_M*Sym_M+enc_S*Sym_S)*enc_M*Sus_M-alpha*lam*f_env_T50(Ef1,k,T_50,h_T)*(enc_Y*Asym_Y+enc_M*Asym_M+enc_S*Asym_S)*enc_M*Sus_M ...
             -alpha*lam*f_env_T50(Ef1,k,T_50,h_T)*Qu*(enc_Y*SymImm_Y+enc_M*SymImm_M+enc_S*SymImm_S)*enc_M*Sus_M-alpha*lam*f_env_T50(Ef1,k,T_50,h_T)*(enc_Y*AsymImm_Y+enc_M*AsymImm_M+enc_S*AsymImm_S)*enc_M*Sus_M ...
            -alpha*lam*f_env_T50(Ef1,k,T_50,h_T)*Qu*Shi_IC*(enc_Y*SymIC_Y+enc_M*SymIC_M+enc_S*SymIC_S)*enc_M*Sus_M...
            -trav*Sus_M-Vac1Sus_M+phi_SusImm*SusImm_M...
            -inIC_M+inIC_M/PopICss_M*SusIC_M;
    dSus_S=-alpha*lam*f_env_T50(Ef1,k,T_50,h_T)*Qu*(enc_Y*Sym_Y+enc_M*Sym_M+enc_S*Sym_S)*enc_S*Sus_S-alpha*lam*f_env_T50(Ef1,k,T_50,h_T)*(enc_Y*Asym_Y+enc_M*Asym_M+enc_S*Asym_S)*enc_S*Sus_S ...
            -alpha*lam*f_env_T50(Ef1,k,T_50,h_T)*Qu*(enc_Y*SymImm_Y+enc_M*SymImm_M+enc_S*SymImm_S)*enc_S*Sus_S-alpha*lam*f_env_T50(Ef1,k,T_50,h_T)*(enc_Y*AsymImm_Y+enc_M*AsymImm_M+enc_S*AsymImm_S)*enc_S*Sus_S ...
         -alpha*lam*f_env_T50(Ef1,k,T_50,h_T)*Qu*Shi_IC*(enc_Y*SymIC_Y+enc_M*SymIC_M+enc_S*SymIC_S)*enc_S*Sus_S...
            -trav*Sus_S-Vac1Sus_S+phi_SusImm*SusImm_S...
        	-inIC_S+inIC_S/PopICss_S*SusIC_S;


    dInc_Y=+alpha*lam*f_env_T50(Ef1,k,T_50,h_T)*Qu*(enc_Y*Sym_Y+enc_M*Sym_M+enc_S*Sym_S)*enc_Y*Sus_Y+alpha*lam*f_env_T50(Ef1,k,T_50,h_T)*(enc_Y*Asym_Y+enc_M*Asym_M+enc_S*Asym_S)*enc_Y*Sus_Y ...
            +alpha*lam*f_env_T50(Ef1,k,T_50,h_T)*Qu*(enc_Y*SymImm_Y+enc_M*SymImm_M+enc_S*SymImm_S)*enc_Y*Sus_Y+alpha*lam*f_env_T50(Ef1,k,T_50,h_T)*(enc_Y*AsymImm_Y+enc_M*AsymImm_M+enc_S*AsymImm_S)*enc_Y*Sus_Y ...
            +alpha*lam*f_env_T50(Ef1,k,T_50,h_T)*Qu*Shi_IC*(enc_Y*SymIC_Y+enc_M*SymIC_M+enc_S*SymIC_S)*enc_Y*Sus_Y...
            +trav*Sus_Y -tau*Inc_Y;
    dInc_M=+alpha*lam*f_env_T50(Ef1,k,T_50,h_T)*Qu*(enc_Y*Sym_Y+enc_M*Sym_M+enc_S*Sym_S)*enc_M*Sus_M+alpha*lam*f_env_T50(Ef1,k,T_50,h_T)*(enc_Y*Asym_Y+enc_M*Asym_M+enc_S*Asym_S)*enc_M*Sus_M ...
             +alpha*lam*f_env_T50(Ef1,k,T_50,h_T)*Qu*(enc_Y*SymImm_Y+enc_M*SymImm_M+enc_S*SymImm_S)*enc_M*Sus_M+alpha*lam*f_env_T50(Ef1,k,T_50,h_T)*(enc_Y*AsymImm_Y+enc_M*AsymImm_M+enc_S*AsymImm_S)*enc_M*Sus_M ...
              +alpha*lam*f_env_T50(Ef1,k,T_50,h_T)*Qu*Shi_IC*(enc_Y*SymIC_Y+enc_M*SymIC_M+enc_S*SymIC_S)*enc_M*Sus_M...
            +trav*Sus_M-tau*Inc_M;
    dInc_S=+alpha*lam*f_env_T50(Ef1,k,T_50,h_T)*Qu*(enc_Y*Sym_Y+enc_M*Sym_M+enc_S*Sym_S)*enc_S*Sus_S+alpha*lam*f_env_T50(Ef1,k,T_50,h_T)*(enc_Y*Asym_Y+enc_M*Asym_M+enc_S*Asym_S)*enc_S*Sus_S ...
            +alpha*lam*f_env_T50(Ef1,k,T_50,h_T)*Qu*(enc_Y*SymImm_Y+enc_M*SymImm_M+enc_S*SymImm_S)*enc_S*Sus_S+alpha*lam*f_env_T50(Ef1,k,T_50,h_T)*(enc_Y*AsymImm_Y+enc_M*AsymImm_M+enc_S*AsymImm_S)*enc_S*Sus_S ...
            +alpha*lam*f_env_T50(Ef1,k,T_50,h_T)*Qu*Shi_IC*(enc_Y*SymIC_Y+enc_M*SymIC_M+enc_S*SymIC_S)*enc_S*Sus_S...
            +trav*Sus_S-tau*Inc_S;


    dSym_Y=tau*Inc_Y*(1-f_Asy)-omega*H_Y*Sym_Y-ro_Sym*Sym_Y;
    dSym_M=tau*Inc_M*(1-f_Asy)-omega*H_M*Sym_M-ro_Sym*Sym_M;
    dSym_S=tau*Inc_S*(1-f_Asy)-omega*H_S*Sym_S-ro_Sym*Sym_S;


    dAsym_Y=tau*Inc_Y*(f_Asy)-ro_Asym*Asym_Y-Vac1Asym_Y;
    dAsym_M=tau*Inc_M*(f_Asy)-ro_Asym*Asym_M-Vac1Asym_M;
    dAsym_S=tau*Inc_S*(f_Asy)-ro_Asym*Asym_S-Vac1Asym_S;

    dHosp_Y=omega*H_Y*Sym_Y-ro_hos*Hosp_Y-delta*D_Y*Hosp_Y;
    dHosp_M=omega*H_M*Sym_M-ro_hos*Hosp_M-delta*D_M*Hosp_M;
    dHosp_S=omega*H_S*Sym_S-ro_hos*Hosp_S-delta*D_S*Hosp_S;

    dResInf_Y=ro_Sym*Sym_Y+ro_Asym*Asym_Y+ro_hos*Hosp_Y-phi_ResInf*ResInf_Y-Vac1ResInf_Y;
    dResInf_M=ro_Sym*Sym_M+ro_Asym*Asym_M+ro_hos*Hosp_M-phi_ResInf*ResInf_M-Vac1ResInf_M;
    dResInf_S=ro_Sym*Sym_S+ro_Asym*Asym_S+ro_hos*Hosp_S-phi_ResInf*ResInf_S-Vac1ResInf_S;

    dDead_Y=delta*D_Y*Hosp_Y;
    dDead_M=delta*D_M*Hosp_M;
    dDead_S=delta*D_S*Hosp_S;


    %Immunized pop

    dSusImm_Y=(effi1_sev-effi1_mild/effi1_sev)*Vac1Sus_Y+(effi1_sev-effi1_mild/effi1_sev)*Vac1Asym_Y ...
            -alpha*lam*f_env_T50(Ef1,k,T_50,h_T)*Qu*(enc_Y*Sym_Y+enc_M*Sym_M+enc_S*Sym_S)*enc_Y*SusImm_Y-alpha*lam*f_env_T50(Ef1,k,T_50,h_T)*(enc_Y*Asym_Y+enc_M*Asym_M+enc_S*Asym_S)*enc_Y*SusImm_Y ...
            -alpha*lam*f_env_T50(Ef1,k,T_50,h_T)*Qu*(enc_Y*SymImm_Y+enc_M*SymImm_M+enc_S*SymImm_S)*enc_Y*SusImm_Y-alpha*lam*f_env_T50(Ef1,k,T_50,h_T)*(enc_Y*AsymImm_Y+enc_M*AsymImm_M+enc_S*AsymImm_S)*enc_Y*SusImm_Y ...
            -alpha*lam*f_env_T50(Ef1,k,T_50,h_T)*Qu*Shi_IC*(enc_Y*SymIC_Y+enc_M*SymIC_M+enc_S*SymIC_S)*enc_Y*SusImm_Y...
            -trav*SusImm_Y+phi_ResInf*ResInf_Y+phi_ResMixed*ResMixed_Y-BoosterSusImm_Y-phi_SusImm*SusImm_Y;
    dSusImm_M=(effi1_sev-effi1_mild/effi1_sev)*Vac1Sus_M+(effi1_sev-effi1_mild/effi1_sev)*Vac1Asym_M ...
            -alpha*lam*f_env_T50(Ef1,k,T_50,h_T)*Qu*(enc_Y*Sym_Y+enc_M*Sym_M+enc_S*Sym_S)*enc_M*SusImm_M-alpha*lam*f_env_T50(Ef1,k,T_50,h_T)*(enc_Y*Asym_Y+enc_M*Asym_M+enc_S*Asym_S)*enc_M*SusImm_M ...
            -alpha*lam*f_env_T50(Ef1,k,T_50,h_T)*Qu*(enc_Y*SymImm_Y+enc_M*SymImm_M+enc_S*SymImm_S)*enc_M*SusImm_M-alpha*lam*f_env_T50(Ef1,k,T_50,h_T)*(enc_Y*AsymImm_Y+enc_M*AsymImm_M+enc_S*AsymImm_S)*enc_M*SusImm_M ...
            -alpha*lam*f_env_T50(Ef1,k,T_50,h_T)*Qu*Shi_IC*(enc_Y*SymIC_Y+enc_M*SymIC_M+enc_S*SymIC_S)*enc_Y*SusImm_M...
            -trav*SusImm_M+phi_ResInf*ResInf_M+phi_ResMixed*ResMixed_M-BoosterSusImm_M-phi_SusImm*SusImm_M;
    dSusImm_S=(effi1_sev-effi1_mild/effi1_sev)*Vac1Sus_S+(effi1_sev-effi1_mild/effi1_sev)*Vac1Asym_S...
            -alpha*lam*f_env_T50(Ef1,k,T_50,h_T)*Qu*(enc_Y*Sym_Y+enc_M*Sym_M+enc_S*Sym_S)*enc_S*SusImm_S-alpha*lam*f_env_T50(Ef1,k,T_50,h_T)*(enc_Y*Asym_Y+enc_M*Asym_M+enc_S*Asym_S)*enc_S*SusImm_S ...
            -alpha*lam*f_env_T50(Ef1,k,T_50,h_T)*Qu*(enc_Y*SymImm_Y+enc_M*SymImm_M+enc_S*SymImm_S)*enc_S*SusImm_S-alpha*lam*f_env_T50(Ef1,k,T_50,h_T)*(enc_Y*AsymImm_Y+enc_M*AsymImm_M+enc_S*AsymImm_S)*enc_S*SusImm_S ...
            -alpha*lam*f_env_T50(Ef1,k,T_50,h_T)*Qu*Shi_IC*(enc_Y*SymIC_Y+enc_M*SymIC_M+enc_S*SymIC_S)*enc_Y*SusImm_S...
            -trav*SusImm_S+phi_ResInf*ResInf_S+phi_ResMixed*ResMixed_S-BoosterSusImm_S-phi_SusImm*SusImm_S;

    dIncImm_Y= +alpha*lam*f_env_T50(Ef1,k,T_50,h_T)*Qu*(enc_Y*Sym_Y+enc_M*Sym_M+enc_S*Sym_S)*enc_Y*SusImm_Y+alpha*lam*f_env_T50(Ef1,k,T_50,h_T)*(enc_Y*Asym_Y+enc_M*Asym_M+enc_S*Asym_S)*enc_Y*SusImm_Y ...
            +alpha*lam*f_env_T50(Ef1,k,T_50,h_T)*Qu*(enc_Y*SymImm_Y+enc_M*SymImm_M+enc_S*SymImm_S)*enc_Y*SusImm_Y+alpha*lam*f_env_T50(Ef1,k,T_50,h_T)*(enc_Y*AsymImm_Y+enc_M*AsymImm_M+enc_S*AsymImm_S)*enc_Y*SusImm_Y ...
            +alpha*lam*f_env_T50(Ef1,k,T_50,h_T)*Qu*Shi_IC*(enc_Y*SymIC_Y+enc_M*SymIC_M+enc_S*SymIC_S)*enc_Y*SusImm_Y...
            +trav*SusImm_Y-tau*IncImm_Y;
    dIncImm_M=+alpha*lam*f_env_T50(Ef1,k,T_50,h_T)*Qu*(enc_Y*Sym_Y+enc_M*Sym_M+enc_S*Sym_S)*enc_M*SusImm_M+alpha*lam*f_env_T50(Ef1,k,T_50,h_T)*(enc_Y*Asym_Y+enc_M*Asym_M+enc_S*Asym_S)*enc_M*SusImm_M ...
            +alpha*lam*f_env_T50(Ef1,k,T_50,h_T)*Qu*(enc_Y*SymImm_Y+enc_M*SymImm_M+enc_S*SymImm_S)*enc_M*SusImm_M+alpha*lam*f_env_T50(Ef1,k,T_50,h_T)*(enc_Y*AsymImm_Y+enc_M*AsymImm_M+enc_S*AsymImm_S)*enc_M*SusImm_M ...
               +alpha*lam*f_env_T50(Ef1,k,T_50,h_T)*Qu*Shi_IC*(enc_Y*SymIC_Y+enc_M*SymIC_M+enc_S*SymIC_S)*enc_Y*SusImm_M...
               +trav*SusImm_M-tau*IncImm_M;
    dIncImm_S=+alpha*lam*f_env_T50(Ef1,k,T_50,h_T)*Qu*(enc_Y*Sym_Y+enc_M*Sym_M+enc_S*Sym_S)*enc_S*SusImm_S+alpha*lam*f_env_T50(Ef1,k,T_50,h_T)*(enc_Y*Asym_Y+enc_M*Asym_M+enc_S*Asym_S)*enc_S*SusImm_S ...
            +alpha*lam*f_env_T50(Ef1,k,T_50,h_T)*Qu*(enc_Y*SymImm_Y+enc_M*SymImm_M+enc_S*SymImm_S)*enc_S*SusImm_S+alpha*lam*f_env_T50(Ef1,k,T_50,h_T)*(enc_Y*AsymImm_Y+enc_M*AsymImm_M+enc_S*AsymImm_S)*enc_S*SusImm_S ...
            +alpha*lam*f_env_T50(Ef1,k,T_50,h_T)*Qu*Shi_IC*(enc_Y*SymIC_Y+enc_M*SymIC_M+enc_S*SymIC_S)*enc_Y*SusImm_S...
            +trav*SusImm_S-tau*IncImm_S;

    dSymImm_Y=tau*IncImm_Y*(1-f_Asy)-ro_Asym*SymImm_Y;
    dSymImm_M=tau*IncImm_M*(1-f_Asy)-ro_Asym*SymImm_M;
    dSymImm_S=tau*IncImm_S*(1-f_Asy)-ro_Asym*SymImm_S;


    dAsymImm_Y=tau*IncImm_Y*(f_Asy)-ro_Asym*AsymImm_Y-BoosterAsymImm_Y;
    dAsymImm_M=tau*IncImm_M*(f_Asy)-ro_Asym*AsymImm_M-BoosterAsymImm_M;
    dAsymImm_S=tau*IncImm_S*(f_Asy)-ro_Asym*AsymImm_S-BoosterAsymImm_S;

    dResMixed_Y=ro_Asym*SymImm_Y+ro_Asym*AsymImm_Y...
            +effi1_mild/effi1_sev*Vac1Sus_Y+effi1_mild/effi1_sev*Vac1Asym_Y+Vac1ResInf_Y...
            +BoosterSusImm_Y+BoosterAsymImm_Y...
            -phi_ResMixed*ResMixed_Y;
    dResMixed_M=ro_Asym*SymImm_M+ro_Asym*AsymImm_M...
        +effi1_mild/effi1_sev*Vac1Sus_M+effi1_mild/effi1_sev*Vac1Asym_M+Vac1ResInf_M+...
        +BoosterSusImm_M+BoosterAsymImm_M...
        -phi_ResMixed*ResMixed_M;
    dResMixed_S=ro_Asym*SymImm_S+ro_Asym*AsymImm_S...
        +effi1_mild/effi1_sev*Vac1Sus_S+Vac1ResInf_S...
        +BoosterSusImm_S+BoosterAsymImm_S...
        -phi_ResMixed*ResMixed_S;


    dNewcases_Y=tau*Inc_Y+tau*IncIC_Y+tau*IncImm_Y;
    dNewcases_M=tau*Inc_M+tau*IncIC_M+tau*IncImm_M;
    dNewcases_S=tau*Inc_S+tau*IncIC_S+tau*IncImm_S;

    dNewcasesIC_Y=tau*IncIC_Y;
    dNewcasesIC_M=tau*IncIC_M;
    dNewcasesIC_S=tau*IncIC_S;

    dAdmin_Y=omega*H_Y*Sym_Y;
    dAdmin_M=omega*H_M*Sym_M;
    dAdmin_S=omega*H_S*Sym_S;
    dAdminIC_Y=omega_IC*HIC_Y*SymIC_Y;
    dAdminIC_M=omega_IC*HIC_M*SymIC_M;
    dAdminIC_S=omega_IC*HIC_S*SymIC_S;

    dydt = [dSus_Y; dSus_M; dSus_S; dInc_Y; dInc_M; dInc_S; dSym_Y; dSym_M; dSym_S; dAsym_Y; dAsym_M; dAsym_S; dHosp_Y; dHosp_M; dHosp_S;dResInf_Y; dResInf_M; dResInf_S;...
    dDead_Y; dDead_M; dDead_S;...
    dSusImm_Y; dSusImm_M; dSusImm_S; dIncImm_Y; dIncImm_M; dIncImm_S; dSymImm_Y; dSymImm_M; dSymImm_S; dAsymImm_Y; dAsymImm_M; dAsymImm_S; dResMixed_Y;  dResMixed_M;  dResMixed_S;...
    dSusIC_Y; dSusIC_M; dSusIC_S; dIncIC_Y; dIncIC_M; dIncIC_S; dSymIC_Y; dSymIC_M; dSymIC_S; dHospIC_Y; dHospIC_M; dHospIC_S;dResIC_Y; dResIC_M; dResIC_S; dDeadIC_Y; dDeadIC_M; dDeadIC_S;...
    dNewcases_Y;dNewcases_M;dNewcases_S; dNewcasesIC_Y;dNewcasesIC_M;dNewcasesIC_S;...
    dAdmin_Y;dAdmin_M;dAdmin_S;dAdminIC_Y;dAdminIC_M;dAdminIC_S];

end

function [X]=f_env_T50(ef,k,T_50,h_T)
  X=k*T_50^h_T/(T_50^h_T+ef^h_T);

end
```

```
Not enough input arguments.

Error in Covid_ImmComprom_ODEs (line 5)
a_0=para(1);%infection rate 
    ^^^^^^^
```

Published with MATLAB® R2024b
