# Supplementary figures and images for: A dynamic model of COVID-19 infection quantifies the impact of preventive interventions on the infection of severely immunocompromised subjects in the United Kingdom
Source: PLoS One. 2026 Feb 23;21(2):e0341331. doi: 10.1371/journal.pone.0341331 (PMC12928435; doi:10.1371/journal.pone.0341331)

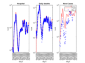

Supplement: S2 File — (ZIP) [file pone.0341331.s002.zip › Covid_ImmComprom_Integration.png]

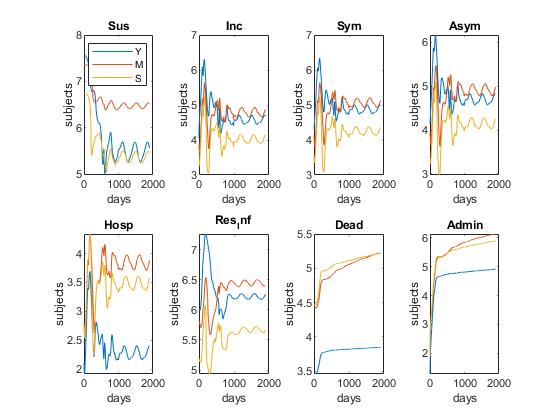

Supplement: S2 File — (ZIP) [file pone.0341331.s002.zip › Covid_ImmComprom_Integration_01.png]

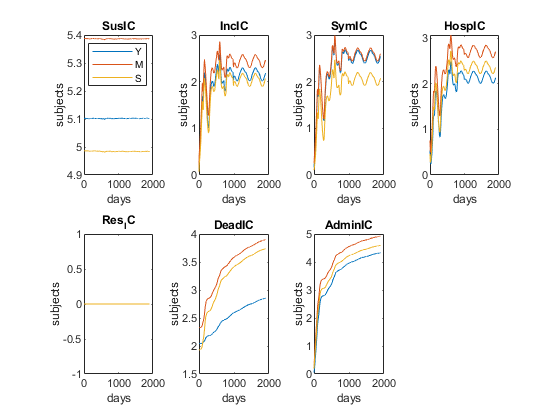

Supplement: S2 File — (ZIP) [file pone.0341331.s002.zip › Covid_ImmComprom_Integration_02.png]

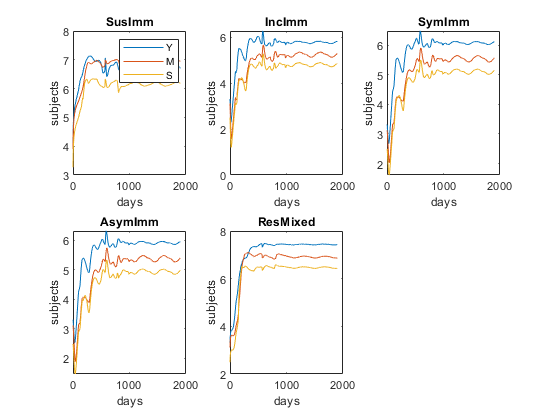

Supplement: S2 File — (ZIP) [file pone.0341331.s002.zip › Covid_ImmComprom_Integration_03.png]

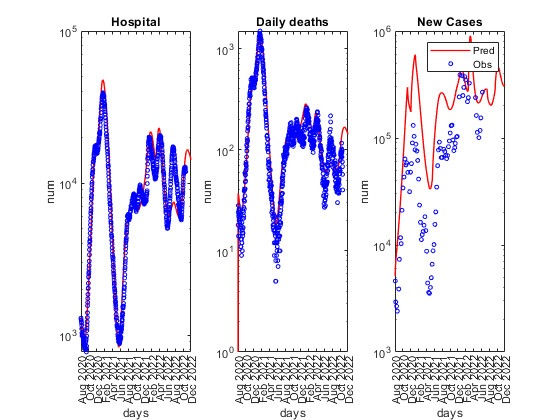

Supplement: S2 File — (ZIP) [file pone.0341331.s002.zip › Covid_ImmComprom_Integration_04.png]
